# Genes Contributing to *Porphyromonas gingivalis* Fitness in Abscess and Epithelial Cell Colonization Environments

**DOI:** 10.3389/fcimb.2017.00378

**Published:** 2017-08-28

**Authors:** Daniel P. Miller, Justin A. Hutcherson, Yan Wang, Zuzanna M. Nowakowska, Jan Potempa, Deborah R. Yoder-Himes, David A. Scott, Marvin Whiteley, Richard J. Lamont

**Affiliations:** ^1^Department of Oral Immunology and Infectious Diseases, University of Louisville Louisville, KY, United States; ^2^Department of Microbiology, Faculty of Biochemistry, Biophysics and Biotechnology, Jagiellonian University Krakow, Poland; ^3^Malopolska Centre of Biotechnology, Jagiellonian University Krakow, Poland; ^4^Department of Biology, University of Louisville Louisville, KY, United States; ^5^Department of Molecular Biosciences, University of Texas at Austin Austin, TX, United States

**Keywords:** Tn-Seq, pathogenicity, periodontal, oral, fitness

## Abstract

*Porphyromonas gingivalis* is an important cause of serious periodontal diseases, and is emerging as a pathogen in several systemic conditions including some forms of cancer. Initial colonization by *P. gingivalis* involves interaction with gingival epithelial cells, and the organism can also access host tissues and spread haematogenously. To better understand the mechanisms underlying these properties, we utilized a highly saturated transposon insertion library of *P. gingivalis*, and assessed the fitness of mutants during epithelial cell colonization and survival in a murine abscess model by high-throughput sequencing (Tn-Seq). Transposon insertions in many genes previously suspected as contributing to virulence showed significant fitness defects in both screening assays. In addition, a number of genes not previously associated with *P. gingivalis* virulence were identified as important for fitness. We further examined fitness defects of four such genes by generating defined mutations. Genes encoding a carbamoyl phosphate synthetase, a replication-associated recombination protein, a nitrosative stress responsive HcpR transcription regulator, and RNase Z, a zinc phosphodiesterase, showed a fitness phenotype in epithelial cell colonization and in a competitive abscess infection. This study verifies the importance of several well-characterized putative virulence factors of *P. gingivalis* and identifies novel fitness determinants of the organism.

## Introduction

*Porphyromonas gingivalis*, an oral anaerobe, is a common constituent of the subgingival microbiota in humans. While the organism is usually in mutualistic balance with the host, breakdown of homeostasis and the induction of dysbiotic host responses leads to chronic periodontal diseases, one of the most common infectious diseases of humans worldwide (Kassebaum et al., 2014; Hajishengallis and Lamont, 2016). *P. gingivalis* is also epidemiologically and physically associated with several serious systemic conditions including arthrosclerosis, rheumatoid arthritis, and some forms of cancer such as oral squamous cell carcinoma (Kumar, 2013; Maddi and Scannapieco, 2013; Whitmore and Lamont, 2014; Atanasova and Yilmaz, 2015). Studies of *P. gingivalis* pathogenicity predominantly have utilized genetic mutation approaches and investigation of host responses (Lamont and Jenkinson, 1998; Aruni et al., 2013; Sakanaka et al., 2016). Through decades of accumulated data, *P. gingivalis* is known to express a plurality of virulence factors including fimbriae, gingipains and other proteases, tetratricopeptide repeat (TPR) motif proteins, extracellular polysaccharide, hemin uptake systems, and LPS, all of which have demonstrated importance in animal models of periodontal disease (Guo et al., 2010; Lewis, 2010; Bostanci and Belibasakis, 2012; Hajishengallis et al., 2015; Lamont and Hajishengallis, 2015; Nakayama, 2015; Shoji and Nakayama, 2016; Smalley and Olczak, 2017). However, despite these advances, we still know little of the genes and gene products that contribute to fitness of the organism in the differing microenvironments of the oral subgingival compartment or in the deeper host tissues.

*P. gingivalis* can colonize the subgingival plaque biofilm in healthy individuals and is well-adapted to thrive in the multispecies biofilm community. However, the initiation and progression of periodontal disease episodes involve a closer interaction with host tissues and with inflammatory responses. The mouse abscess model has been widely used as a screen for *P. gingivalis* virulence factors required for survival in an *in vivo* environment (Graves et al., 2008; Hajishengallis et al., 2015). Components of *P. gingivalis* found to be important in this system include gingipains (Yoneda et al., 2001), fimbriae (Nakano et al., 2004), and TprA, a tetratricopeptide repeat protein (Kondo et al., 2010). Moreover, resistance to oxidative stress can protect against oxidative killing within professional phagocytic cells and contribute to *in vivo* survival (Olsen and Hajishengallis, 2016; Sochalska and Potempa, 2017).

On the oral mucosal membranes, *P. gingivalis* can engage the epithelial cells of the periodontal pocket in an interactive dialog that results in internalization and intracellular survival of the organism (Lamont et al., 1995; Yilmaz, 2008; Tribble and Lamont, 2010; Bostanci and Belibasakis, 2012). The mechanics of *P. gingivalis* internalization within gingival epithelial cells have been investigated in detail. The FimA-component fimbriae of *P. gingivalis* mediate attachment to β1 integrins on the epithelial cell surface with subsequent activation of integrin-dependent signaling components including the focal adhesion protein paxillin (Yilmaz et al., 2002). Additionally *P. gingivalis* secretes a serine phosphatase, SerB, which can locate intracellularly where it dephosphorylates and activates the actin depolymerizing host protein cofilin (Tribble et al., 2006; Moffatt et al., 2012). The counteracting functions of cofilin-dependent depolymerization, and integrin-dependent polymerization of actin result in transient rearrangement of the microfilament cytoskeleton which facilitates entry of *P. gingivalis*. However, *P. gingivalis* strains that are mutant in *fimA* or *serB* are still capable of invasion, albeit at lower levels (Yilmaz et al., 2003; Tribble et al., 2006), indicating the existence of additional unidentified *P. gingivalis* functions that contribute to internalization and intracellular survival. Intracellular *P. gingivalis* are protected from immune mediators and from antibiotics, and can serve as a source for recrudescence of infection following physical removal of pathogenic biofilms (Johnson et al., 2008; Tribble and Lamont, 2010). Strains of *P. gingivalis* isolated from chronic infection tend to be more invasive than strains isolated from healthy sites, consistent with an important role for epithelial cell internalization in the disease process (Jandik et al., 2008; Baek et al., 2015).

*P. gingivalis* expresses two different LPS molecules, O-LPS and A-LPS (or APS) (Shoji and Nakayama, 2016). O-LPS can exist in different isoforms which act either as an agonist or antagonist of TLR signaling, depending on the pattern of acylation and phosphorylation (Coats et al., 2009). A-LPS is a phosphorylated branched mannan (Paramonov et al., 2005), that is attached to many proteins translocated through the type IX section system (T9SS), thus anchoring them to the bacterial surface (De Diego et al., 2016). A mutant unable to incorporate A-LPS into T9SS substrates is less virulent in the mouse subcutaneous infection model (Taguchi et al., 2015). In addition to LPS, *P. gingivalis* synthesizes a variety of novel membrane lipids, including species of dihydroceramide sphingolipids (Nichols et al., 2004, 2012; Moye et al., 2016). Sphingolipids play an essential role in long-term survival of *P. gingivalis* and in resistance to oxidative stress. In the absence of sphingolipds, membrane perturbations disrupt linkage of gingipains to the cell surface, and modulate the presentation of surface polysaccharides (Moye et al., 2016).

*P. gingivalis* strains produce K-antigen extracellular polysaccharide which can be organized into a capsule (Laine et al., 1997) or more diffusely secreted (Maeda et al., 2008). Encapsulated strains are more resistant to phagocytosis (Singh et al., 2011) and cause a spreading infection in the mouse subcutaneous infection model (Laine and Van Winkelhoff, 1998). In addition, capsule-dependent coaggregation with *Fusobacterium nucleatum* led to increased invasion of *P. gingivalis* into epithelial cells and more severe periodontitis in a murine model (Polak et al., 2017), However, the presence of capsule may be detrimental to invasion of monocultures of *P. gingivalis* into host cells (Irshad et al., 2012). Production of extracellular polysaccharide can be controlled by a tyrosine phosphatase (Ltp1) and a tyrosine kinase (Ptk1) (Maeda et al., 2008; Wright et al., 2014) which participate in a secretion system homologous to the Wzy-dependent mechanism in *E. coli* (Whitfield, 2006). In addition, expression of genes involved in both K-antigen and A-LPS synthesis can be controlled by the DNABII protein HU β-subunit (Alberti-Segui et al., 2010; Priyadarshini et al., 2013) and an antisense RNA (asRNA) molecule located within a 77-bp inverted repeat (77bpIR) element located near the 5′ end of the K-antigen locus (Bainbridge et al., 2015). DNABII proteins are also important in maintaining the structure of the eDNA component of the extracellular polymeric substance (EPS) in *P. gingivalis* biofilms (Rocco et al., 2017).

Transposon mutagenesis combined with high throughput sequencing (Tn-Seq) is now a commonly used technique to study the fitness of bacteria under different selective pressures (Valentino et al., 2014; Gutierrez et al., 2015; Troy et al., 2016). Transposon mutagenesis can create a large pool of highly-saturated mutant libraries, and the comparative contributions of bacterial genes can be assessed following selection for fitness in different environments. In this study, we utilized a *P. gingivalis* Mariner based Tn library (Hutcherson et al., 2016) to perform an unbiased search for genes involved in epithelial cell interactions and *in vivo* survival in a murine abscess model. Genes encoding previously unrecognized properties that make measurable contributions to survival in these contexts were selected for further analysis by targeted gene disruption. The results show that genes encoding many well-characterized potential virulence determinants were essential under these conditions. In addition, several novel fitness determinants were identified.

## Methods

### Bacterial and eukaryotic cell culture

*P. gingivalis* strain ATCC 33277 (33277) was cultured in GAM (Gifu anaerobic medium) anaerobically at 37°C. For solid culture, GAM agar plates were supplemented with defibrinated sheep's blood. Isogenic mutants, ΔPGN_0770, ΔPGN_1200, ΔPGN_1300, and ΔPGN_1444, were grown with either 1 μg/ml of tetracycline or 5 μg/ml of erythromycin. The transposon libraries were maintained in GAM containing 50 μg/ml of gentamicin and 5 μg/ml of erythromycin. Human telomerase immortalized keratinocytes (TIGKs) derived from gingival epithelium were cultured at 37°C and 5% CO_2_ in Dermalife-K serum-free culture medium (Lifeline Cell Technology, Carlsbad, CA) as described (Moffatt-Jauregui et al., 2013). TIGKs were used at passage 20 and at 80% confluence.

### *P. gingivalis* transposon library

The construction of the transposon library was previously described by Hutcherson et al. (2016). Briefly, a saturated transposon library was generated using a mariner transposon system (Goodman et al., 2009) in *P. gingivalis* 33277. The constructed library was passaged in GAM with antibiotics, and this input library was aliquoted at 10^10^ CFU and stored at −80°C.

### *In vitro* epithelial cell colonization screen

The *P. gingivalis* transposon library was cultured to optical density (OD)_600_ 1.0 and added to TIGK cells (12-fold replicates) at a multiplicity of infection (MOI) of 10. At this MOI, *P. gingivalis* exhibits high levels of invasion (over 5%) and attachment (Lamont et al., 1995; Capestany et al., 2008). After 30 min, the supernatant was removed and the cells washed twice with phosphate buffered saline (PBS), and removed by scraping. Cells were lysed by sonication, and after centrifugation the pellets were resuspended in GAM with gentamicin and erythromycin, and incubated anaerobically for 3–4 days. When OD >1.0 was reached, the cell infection procedure was repeated. After the second round of infections, bacteria grown in GAM were stored in aliquots at −80°C for use as the TIGK output library.

### *In vivo* mouse abscess screen

All experiments with mice were reviewed and approved by the University of Louisville Institutional Animal Care and Use Committee. Balb/c mice, 8–10 weeks old, were inoculated dorsally with the *P. gingivalis* transposon library at a concentration of 3 × 10^9^ colony forming units (CFU) in 100 μl PBS. Mice were monitored daily up to 2 weeks. Mice that developed abscesses were euthanized, and the abscess was harvested in sterile PBS. The abscesses were cultured individually in GAM with gentamicin and erythromycin and then pooled at OD 1.0 and cultured for an additional 3–4 days until reaching OD 1.0. This culture was used to prepare the inoculum for a second round of mouse selection. The abscesses were pooled, cultured to OD 1.0, and aliquots were stored at −80°C for use as the mouse output library.

### Construction and sequencing of DNA libraries

Libraries for sequencing were constructed as described previously (Hutcherson et al., 2016). Double-stranded, barcoded, DNA adapters were created using the LIB_Adapt primers (_control, _TIGK or _abscess) (Supplementary Table 1) to differentiate sequencing groups in the same flow lane. Adapters were ligated to gel-purified DNA products using T4 DNA ligase. Ligation products were purified by a Wizard purification kit (Promega) and amplified by PCR using HiFi Hotstart SuperMix with LIB_PCR_5 and LIB_PCR_3 primers (Supplementary Table 1). Products were quantified using a NanoDrop ND-1000 spectrophotometer, and sequenced on an Illumina HiSeq2000 platform at the University of Michigan Core Facility as 50-bp single end reads.

### Sequencing data analysis

Sequencing reads were analyzed by sorting based on barcodes using a custom script in Java and then by CLC Genomics Workbench V7.2 for bioinformatics. Reads were trimmed to remove adapter and transposon sequences, reads that mapped to multiple locations, and sequences with reads with a quality score <0.05 or of <15 nucleotides. The remaining reads were aligned to the annotated gene list of *P. gingivalis* strain 33277. Characteristics of the library are provided in Supplementary Table 2. Reads were counted and normalized to reads per kilobase of transcript per million reads mapped (RPKM), and genes with a RPKM <5 were considered inherently essential (Klein et al., 2012; Hutcherson et al., 2016) and not considered further. The number of reads for each gene in the input pool (i.e., the library of *P. gingivalis* transposon mutants used to inoculate TIGKs or mice) was compared to the number of reads in the equivalent gene in the two output pools (i.e., the library of transposon mutants recovered after selection in mice or in TIGKs) to calculate fold change. Significance was assessed using CLC Genomics Workbench calculated Bonferroni multiple testing correction. Genes with ≥200 reads in the input pool, a ≥10-fold change between the input and output groups, and with a Bonferroni-adjusted *p*-value of less than 0.05 were considered significant for fitness. The individual gene reads within each ORF were also determined, an example of which is shown in Supplementary Figure 1.

### Mutant construction

The PCR fusion technique was utilized to generate allelic exchange mutants of genes identified in output Tn-Seq libraries, as described previously (Simionato et al., 2006), and using the primers listed in Supplementary Table 1. Constructs were introduced into *P. gingivalis* by electroporation and the correct insertion confirmed by PCR and sequencing. There was no difference between parent and any of the mutant strains in growth rate in GAM medium, or in survival in the TIGK cell culture medium.

### Attachment and invasion assays

For attachment (Capestany et al., 2008), TIGKs were cultured in 96-well plates, fixed with 5% buffered formalin for 1 h, and washed with PBS. Cells were reacted with *P. gingivalis* strains at MOI 10 for 30 min at 37°C, and then washed with PBS to remove non-adherent bacteria. Wells were incubated with *P. gingivalis* whole-cell antibodies 1:10,000 at 37°C for 1 h, then washed with PBS. Binding was detected with a secondary horse radish peroxidase (HRP)-anti-rabbit antibody (1:5,000) and 3,3′,5,5′-tetramethylbenzidine substrate (Sigma), and recorded at 450 nm.

For invasion (Lamont et al., 1995), TIGK cells in 24-well plates were reacted with *P. gingivalis* strains at MOI 100 for 1 h at 37°C. The supernatant was removed and wells were washed with PBS. External adherent, non-invaded bacteria were killed by incubation with 300 μg/ml gentamicin and 200 μg/ml metronidazole for 1 h. Cells were lysed with sterile H_2_O, serially diluted in pre-reduced PBS and plated on GAM for viable counting. ANOVA tests were used to determine significance in attachment and invasion assays.

### *In vivo* competitive assay

Balb/c mice were dorsally injected with equal numbers (1.5 × 10^9^) of *P. gingivalis* 33277 and of the respective mutant strain. Mice were monitored daily and abscesses were collected 4–5 days post-infection. DNA was isolated using a DNA wizard kit (Promega), and amplified by qPCR with primers to 33277 16S rRNA or the appropriate antibiotic resistance gene (Supplementary Table 1). Numbers of *P. gingivalis* were calculated by comparison with a standard curve derived from known amounts of *P. gingivalis* or the respective mutant using 16S rRNA and antibiotic resistant primers. Competitive index (CI) was calculated as the output ratio of mutant to parent divided by the input ratio of mutant to parent, and significance determined by the Wilcoxon signed rank test.

## Results and discussion

### Tn-Seq models of fitness

**Figure 1:**
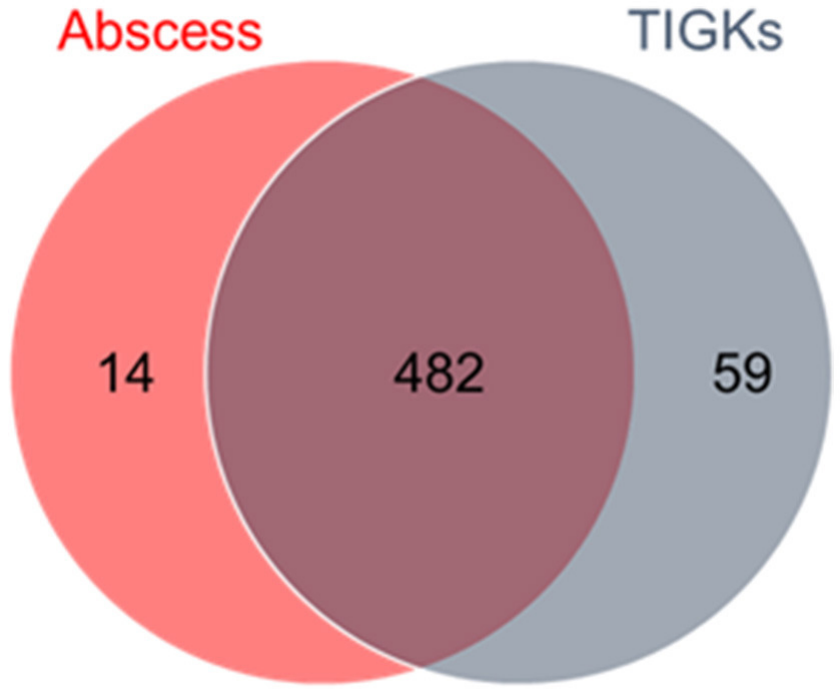
Distribution of essential genes of *P. gingivalis* between selection pressures. Output pools of transposon mutants recovered from the murine abscess and epithelial cell environments were analyzed for negatively selected genes (> log_2_ 3.3 reduction compared to the Tn-Seq library input pool). There were 482 common genes that were selected against in both conditions.

The goal of this study was to identify genes required for epithelial cell colonization and *in vivo* survival by *P. gingivalis*. A previously constructed 80,000 colony Tn-Seq library was tested for fitness in a gingival epithelial cell (TIGK) culture model and a murine abscess model. The epithelial model will identify mutants with a diminished ability to adhere and/or invade and survive within TIGKs. It is also possible that mutants that are less able to survive in the culture medium will be negatively selected; however this is less likely as the assay time was restricted to 30 min and *P. gingivalis* invasion is complete within 15–20 min (Belton et al., 1999). The abscess model will identify mutants with a deficiency in survival in a more complex environment which contains host immune factors. Core essential genes required for *in vitro* growth of *P. gingivalis* have been identified in our previous study (Hutcherson et al., 2016), and these were not considered in the data analysis. In the epithelial cell colonization model, 498 genes were determined conditionally essential, whereas 545 genes were determined essential in the abscess group, using a stringent log2 cutoff of >3.3 which represents a 10-fold difference. The majority of genes (482) were common between the two selection conditions as shown in Figure 1. The concordance between the two selection conditions indicates that both are providing an accurate report of *in vivo* fitness. The genome of *P. gingivalis* strain 33277 contains 2,090 annotated genes (Naito et al., 2008), and thus around a quarter of these make significant contributions to survival in host environments. The large number of conditionally essential genes is consistent with the characterization of *P. gingivalis* as a host adapted organism with a longstanding evolutionary relationship with the host (Tribble et al., 2013; Nadkarni et al., 2014). The contribution of each gene in *P. gingivalis* to fitness is shown in Supplementary Table 3, and the genes fulfilling the criteria as essential in both selection screens are listed in Supplementary Table 4.

### Analysis of genes important for epithelial colonization and *in vivo* survival

#### Metabolism

Conditionally essential genes were imported into Kyoto Encyclopedia of Genes and Genomes (KEGG) and metabolic pathways were queried for the essential genes. While 55 of the 482 genes were annotated as metabolic, these did not differentially populate any metabolic pathway represented in KEGG. These results indicate that in host environments *P. gingivalis* can utilize multiple redundant metabolic pathways, and loss of any one does not confer a fitness disadvantage. On the other hand, functional annotation characterization of essential genes revealed those associated with transport and binding proteins, the cell envelope, and protein fate were enriched.

#### Adhesion

**Figure 2:**
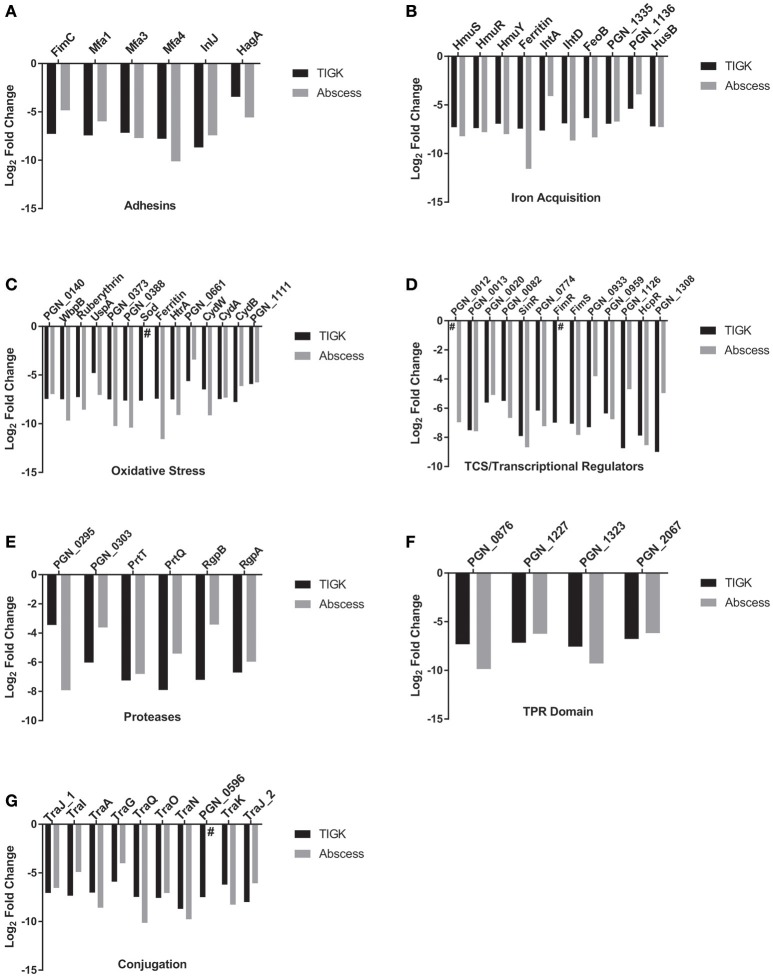
Major functional categories affecting fitness in abscess and TIGK colonization models. Genes affecting fitness of *P. gingivalis* include **(A)** adhesins, **(B)** iron acquisition, **(C)** oxidative stress, **(D)** TCS and transcriptional regulators, **(E)** proteases, **(F)** Tetratricopeptide repeat (TPR) motif proteins and **(G)** conjugation. # indicates that the gene was not detected in the output pool. Gene categories were obtained from http://www.genome.jp/kegg/pathway.html or from previous articles.

To colonize the oral cavity in which there is fluid flow and shear forces, bacteria attach to biotic and abiotic oral surfaces. *P. gingivalis* possess a multiplicity of adhesins including the FimA- and Mfa1- component fimbriae, haemagglutinin (Hag)A, HagB, and HagC, and the leucine-rich repeat domain Internalin InlJ (Lamont and Jenkinson, 2000; Capestany et al., 2006; Kuboniwa and Lamont, 2010; Wright et al., 2013). Of the genes encoding these adhesins, *mfa1, hagA*, and *inlJ* were negatively selected at least 10-fold, as were genes for the Mfa fimbriae accessory proteins Mfa3 and Mfa4 (Figure 2). The gene encoding the major fimbrial structure, *fimA*, had a RPKM <4 and was not included in the analysis. It is unclear if underrepresentation of this gene in the input pool is due to a transposon insertion “cold-spot,” or whether loss of FimA renders the strain less fit in a mixed population with fimbriated cells. Work is ongoing to resolve this issue. The potential relevance of the FimA fimbrial structure is suggested by the observation that *fimC*, encoding an adhesive accessory protein (Pierce et al., 2009), was negatively selected. Moreover, the FimS/R two component system (TCS), which controls transcription across the *fim* operon was also negatively selected (discussed further below).

The Mfa fimbriae mediate attachment to other oral biofilm bacteria, in particular the accessory pathogen *S. gordonii* (Wright et al., 2013), and contribute to auto-aggregation and monotypic biofilm formation (Umemoto and Hamada, 2003; Kuboniwa et al., 2009a). Moreover, strains of *P. gingivalis* with biofilm-forming capacity have been found to be more aggressive in inducing abscesses (Clais et al., 2014). Involvement in epithelial cell colonization, may arise from the ability of Mfa fimbriae to mediate adherence to host cells (Kuboniwa and Lamont, 2010). Mfa1 can also selectively engage the dendritic cell (DC) C-type lectin DC-SIGN, leading to evasion of antibacterial autophagy and lysosome fusion, and intracellular persistence in myeloid DCs (Arjunan et al., 2016), properties that may contribute to survival *in vivo*. The functional roles of the Mfa3 and Mfa4 proteins have yet to be defined; however, Mfa3 is located at the fimbrial tip and is required for integration of Mfa4 and Mfa5 (Hasegawa et al., 2013), and Mfa4 may be necessary for the stability of the fimbrial structure (Ikai et al., 2015).

InlJ has been shown to be involved in adherence to abiotic surfaces (Capestany et al., 2006), and although InlJ is not required for epithelial cell internalization, InlJ protein expression is upregulated in *P. gingivalis* following contact with epithelial cells (Zhang et al., 2005). In *Listeria*, InlJ is a sortase-LPXTG anchored adhesin which is upregulated during infection *in vivo* (Sabet et al., 2008). Listerial InlJ can bind to a variety of human cells *in vitro* (Linden et al., 2008; Sabet et al., 2008), and oral colonization of mice with an *inlJ* mutant results in reduced *Listeria* levels compared to the parental strain (Sabet et al., 2005). The results of the current study indicate that InlJ is also important for epithelial colonization and *vivo* survival of *P. gingivalis*.

HagA is a large protein with a predicted molecular mass of 283.3 kDa and containing multiple contiguous direct repeats of 440–456 amino acids, each of which has hemagglutinin activity (Han et al., 1996). HagA can promote attachment to both epithelial and endothelial cells, and antibodies to the hemagglutinin domain are protective in animal models of oral infection (Frazer et al., 2006; Belanger et al., 2012). Our finding that disruption of *hagA* was deleterious for survival in epithelial cells and murine abscesses is consistent with the documented properties of HagA, and suggest a role for this protein in *P. gingivalis* pathogenicity. On the contrary, while HagB and HagC can also mediate attachment to host cells (Song et al., 2005), and HagB is considered a major virulence factor of the organism (Pingel et al., 2008), mutation in *hagB* or *hagC* did not diminish fitness in our infection models.

#### Iron acquisition

*P. gingivalis* exhibits a strong preference for iron in the form of hemin-containing compounds (Lewis, 2010; Smalley and Olczak, 2017). Consequently, multiple hemin uptake systems with differing affinities and specificities are present in the organism. In addition, *P. gingivalis* also possesses a functional ferrous iron transporter, FeoB (Dashper et al., 2005; Anaya-Bergman et al., 2015). Uptake mechanisms that impacted fitness included the Hmu (Lewis et al., 2006), Iht (Slakeski et al., 2000), and Hus (Gao et al., 2010) systems, and PGN_1335-PGN_1336, a proposed hemin uptake system composed of a surface lipoprotein and an outer membrane TonB-dependent receptor (Anaya-Bergman et al., 2015) (Figure 2). *feoB*, and PGN_0604, the gene encoding the iron storage protein ferritin, were also selected negatively. Hemin can enhance virulence of *P. gingivalis* in animal models (McKee et al., 1986), and loss of FeoB renders *P. gingivalis* avirulent *in vivo* (Dashper et al., 2005). Moreover, hemin levels affect the structure of LPS and its properties as a TLR4 antagonist or antagonist (Al-Qutub et al., 2006). A HmuR-deficient mutant of *P. gingivalis* has been shown to be deficient in multispecies community formation (Kuboniwa et al., 2009b), and a relationship between iron regulation and epithelial colonization has also been established, as mutation of the *P. gingivalis* Fur homolog showed significantly weaker adherence and invasion of epithelial cells (Ciuraszkiewicz et al., 2014). In addition, mutation of genes encoding PGN_1335-PGN_1336 reduces survival of *P. gingivalis* within epithelial cells (Anaya-Bergman et al., 2015). Collectively, the current results and the existing literature show hemin and inorganic iron uptake to be fundamental to fitness.

#### Stress responses

*P. gingivalis* is adapted to the environment of a polymicrobial biofilm (Kuboniwa et al., 2009b; Hendrickson et al., 2017), and intrusion of host tissues can imposes stress on the organism, in particular oxidative stress (Park et al., 2004; Xia et al., 2007). General stress response mechanisms including Heat Shock Proteins and the Clp system were not found to be important for either epithelial colonization or *in vivo* survival. However, transposon disruption of many oxidative stress resistance associated genes was detrimental to fitness in our screens (Figure 2); although the possibility the *P. gingivalis* experienced selective oxidative stress during the transition to cell culture or in the preparation of the inoculum for mouse infection can not be entirely eliminated. While many of these genes comprise the regulon controlled by OxyR a redox-sensitive transcriptional regulator (Diaz et al., 2006), *oxyR* itself was not negatively selected in our analysis, indicating a complex control mechanism for oxidative stress in *P. gingivalis*. Intracellular iron/hemin and oxidative stress are also interconnected as free iron and hydrogen peroxide produce reactive oxidative species through Fenton chemistry (Winterbourn, 1995). In addition, a hemin-limited growth environment significantly enhances OxyR activity (Xie and Zheng, 2012), and μ-oxo bisheme, a cell surface layer of the dimeric heme, protects *P. gingivalis* against H_2_O_2_ (Smalley and Olczak, 2017). Nitrosative stress is discussed further below.

#### Two component systems (TCS) and transcriptional regulators

Bacteria utilize TCS sense environmental conditions and respond with an appropriate transcriptional program (Goulian, 2010). *P. gingivalis* possess a limited number of TCS, six in 33277 along with the hybrid GppX and the orphan response regulator (RR) RprY (Naito et al., 2008). Among the TCS, disruption of genes encoding PGN_0012/PGN_0013 and FimS/FimR, along with the response regulator PGN_0774, reduced fitness in our model systems (Figure 2). As mentioned above, the FimS/R TCS controls transcription across the *fim* operon (Nishikawa and Duncan, 2010), consistent with a role for the FimA-fimbriae in fitness. However, a transcriptome analysis revealed that inactivation of *fimS* resulted in the differential expression of 10% of the *P. gingivalis* genome, including genes encoding seven different transcriptional regulators, and three extracytoplasmic sigma factor genes, (Lo et al., 2010), and so FimS/R could effect fitness independently of FimA expression. FimS/FimR can also control expression of the Mfa fimbriae (Wu et al., 2007) which were identified as necessary for fitness in the current study.

In addition to TCS RRs, strain 33277 contains 21 annotated transcriptional regulators, of which six were negatively selected (Figure 2). These are mostly of unknown function; however, SinR is a negative regulator of polysaccharide production in *P. gingivalis* monospecies biofilms (Yamamoto et al., 2013). PGN_1300 (HcpR) is discussed further below.

#### Extracellular polysaccharide

Extracellular polysaccharides of *P. gingivalis* are controlled by a complex multilevel regulatory system (Bainbridge et al., 2015). Such regulation may be necessary for the context-dependent coordination of polysaccharide levels, as capsule production can impede attachment and initial colonization of *P. gingivalis* (Davey and Duncan, 2006; Irshad et al., 2012), but is important for survival and resistance to killing by host immune cells (Singh et al., 2011). In addition to the polysaccharide production regulator SinR, genes PGN_0223 to PGN_0229, which encode proteins involved in both LPS and surface polysaccharide synthesis (Aduse-Opoku et al., 2006; Bainbridge et al., 2015), were identified as conditionally essential for fitness. Mutation of PGN_0223 results in a shortened O antigen and a significant increase in monospecies biofilm formation (Nakao et al., 2006). While *P. gingivalis* strain 33277 does not produce a capsule (Laine and Van Winkelhoff, 1998) it does produce disorganized extracellular polysaccharide (Maeda et al., 2008). Hence, these data would indicate that the presence of polysaccharide, and not capsule *per se*, is essential for fitness, at least subsequent to attachment and biofilm formation.

#### Proteolytic activity

As an asaccharolytic organism, *P. gingivalis* relies on proteolytic activity to produce peptides from proteins as both a carbon and nitrogen source (Lamont and Jenkinson, 1998; Guo et al., 2010). A number of proteinases are thus produced by the organism, and a class of cysteine proteases, the gingipains, are considered of primary importance in virulence. Gingipains can have arginine (RgpA, RgpB) or lysine (Kgp) specificity, and gingipains account for a large proportion of the extracellular proteolytic activity of *P. gingivalis* (Potempa et al., 1997; Guo et al., 2010). RgpA and Kgp also possess haemagglutinin domains homologous to those of HagA (Potempa et al., 1997; Lamont and Jenkinson, 1998; Fitzpatrick et al., 2009; Guo et al., 2010). In addition to provision of nutritional substrates, gingipains are involved in processing of cell surface proteins and degradation of host molecules including immune effectors and matrix components. Gingipains released within host cells can also degrade host cell signaling molecules (Zhou et al., 2015; Barth and Genco, 2016). Mutants of *P. gingivalis* that are deficient in gingipain production display attenuated virulence in animal models, and antibodies to gingipains are protective in these *in vivo* models (O'Brien-Simpson et al., 2000, 2001; Kuboniwa et al., 2001; Pathirana et al., 2007; Wilensky et al., 2013). Mutations in the arginine-specific protease genes *rgpA* and *rgpB*, were identified in our assays (Figure 2). It is important to note here that RgpA and RgpB can be present in the periplasm and on the cell surface, as well as secreted extracellularly (Potempa et al., 1997; Veillard et al., 2013). Further, a feature of competitive fitness assays is the loss of an extracellular function by one mutant can be compensated in trans by other mutants that retain the property. Collectively, this would indicate that it is the periplasmic and cell-associated activities of RgpA and RgpB that make a significant contribution to fitness. Interestingly, although Kgp is thought to make a more significant contribution to pathogenicity than RgpA/B (De Diego et al., 2014), disruption of *kgp* did not reduce fitness in our models. Kgp plays a less important role in surface protein processing compared to RgpA/B (Kadowaki et al., 1998), and hence this role in maintaining surface integrity may be the most important contribution of the gingipains to fitness. Other proteinases identified were PrtQ and the trypsin-like protease PrtT which is involved in pathogenicity in the murine lesion model (Kesavalu et al., 1996).

#### Tetratricopeptide repeat proteins

The tetratricopeptide repeat (TPR) motif is a protein-protein interaction module found in multiple copies in a variety of functionally different proteins (Cerveny et al., 2013). In *P. gingivalis*, loss of the TPR protein TprA renders the organism less virulent in the murine subcutaneous model of infection (Kondo et al., 2010). TprA interacts with TapA, TapB, and TapC, and this complex has been shown to be cooperatively involved in abscess formation (Kondo et al., 2010). Disruption of *tprA* along with *tapA* (PGN_0152) negatively impacted fitness in both of our model systems, consistent with a role for the associated proteins in both abscess formation and epithelial cell colonization. PGN_1227, PGN_1323, and PGN_2067, which encode additional TPR motif proteins, were also selected negatively (Figure 2). The functions of these proteins have yet to be determined; however PGN_1227 expression is increased in communities of *P. gingivalis* with *S. gordonii* (Hendrickson et al., 2017).

#### Conjugation

*P. gingivalis* strains including 33277 can conjugally transfer both chromosomal DNA and conjugative transposons through the action of *tra* gene homologs of the type IV secretion system (Tribble et al., 2007). Strain 33277 contains three clusters of *tra* genes with more than one ortholog of many of the components. Mutants in *traA, traG, traI, traJ, traK, traN, traO, traQ* all exhibited reduced fitness for epithelial colonization and *in vivo* survival (Figure 2). These data would indicate that adaptation through horizontal gene transfer is an important process for survival of *P. gingivalis* in host environments.

#### Novel determinants of fitness

**Figure 3:**
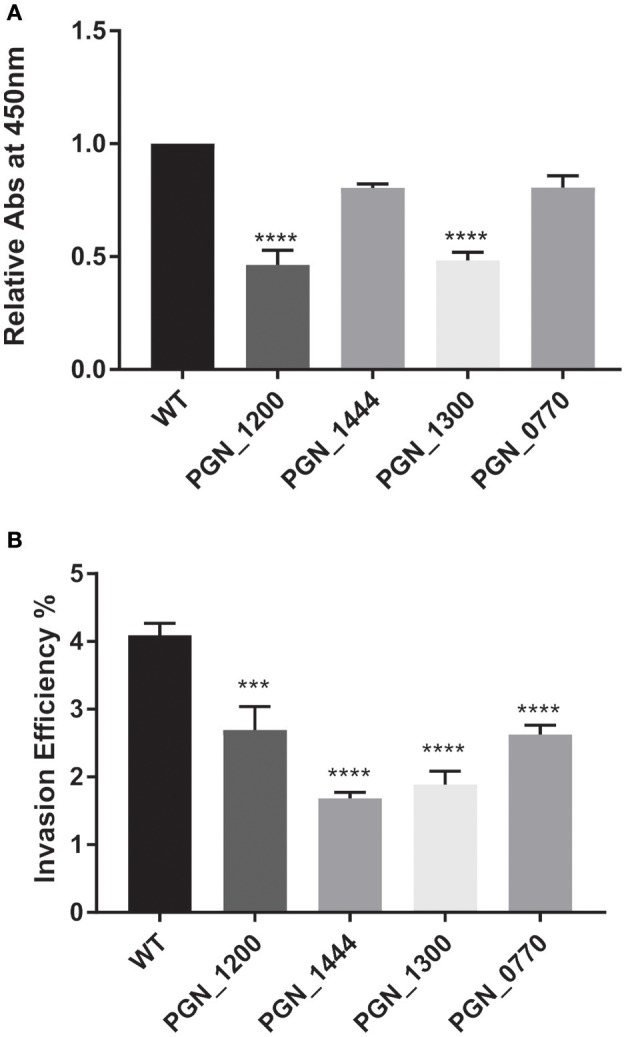
Fitness determinants attenuated in epithelial cell attachment and/or invasion. Genetically defined mutations were constructed in the genes indicated on the x-axis and compared to the parental strain (WT). **(A)** Attachment to formalin-fixed whole TIGK cells was determined in an ELISA assay with *P. gingivalis* antibodies. **(B)** Invasion of TIGK cells was determined by an antibiotic protein assay and intracellular CFU expressed as a percent of input bacterial number. Graphs show mean with standard error of the mean of three independent experiments. ^***^P < 0.01, ^****^P < 0.001 compared to WT by ANOVA with Tukey correction.

**Figure 4:**
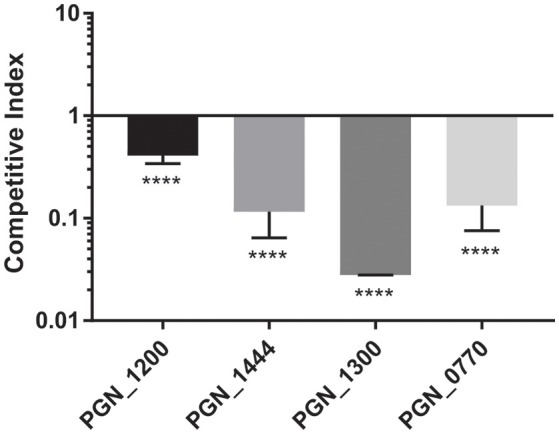
Fitness determinants attenuated during *in vivo* survival competition assays. Genetically defined mutations were constructed in the genes indicated on the x-axis and co-infected in equal numbers with the parental strain in the dorsum of Balb/c female mice. Abscesses were collected 3–4 days post-infection, and numbers of bacteria determined by qPCR. Horizontal line represents a CI of 1. Bars represent mean ± *SD* of one representative experiment of at least three biological replicates. ^****^P < 0.001.

To provide additional insights into *P. gingivalis* fitness, and to partially corroborate the Tn-Seq dataset, specific allelic replacements were constructed in four genes that were operationally essential for fitness. These genes encoded proteins representing major functional classes including metabolic enzymes (PGN_1444), transcriptional regulators (PGN_1300), RNA processing (PGN_0770), and genome stability (PGN_1200). PGN_1444 was the most strongly negatively selected gene in the *in vivo* mouse abscess model, while PGN_0770 and PGN_1200 were the third and fourth most negatively selected in the epithelial cell colonization model. PGN_1300 was negatively selected over 200-fold in both assays. The specific mutants phenocopied the Tn-Seq library results and showed a defect for epithelial colonization as well as a reduced competitive index in the mouse abscess model (Figures 3, 4). Dissection of adherence and invasion properties, further showed that PGN_0770 and PGN_1444 were dispensable for gingival epithelial cell attachment, but were necessary for internalization/intracellular survival (Figure 3).

PGN_1444 is annotated as a carbamoyl phosphate synthetase, an enzyme that catalyzes the ATP-dependent synthesis of carbamoyl phosphate from glutamine or ammonia, an important early step in the synthesis of pyrimidine and citrulline as a precursor of arginine (Cunin et al., 1986). The catabolism of arginine is an important metabolic pathway for *P. gingivalis* (Masuda et al., 2002), and arginine in the culture medium increases fimbrial expression and monotypic biofilm formation (Cugini et al., 2013). In *Streptococcus pneumoniae*, lack of carbamoyl phosphate synthase activity resulted in reduced ability to release NO and H_2_O_2_ (Hoffmann et al., 2006). Moreover, in *Francisella tularensis* carbamoyl phosphate synthetase is required for inhibition of the neutrophil respiratory burst and for intramacrophage growth (Schulert et al., 2009). Hence, while the subject requires further study, PGN_1444 may contribute to *P. gingivalis* fitness through generating arginine that can be used for growth and as a metabolic cue for biofilm formation, and by increasing resistance to the host professional phagocytes.

PGN_1200 is annotated as a Replication-associated recombination protein MgsA/RarA (DNA-dependent ATPase). This protein, along with RecA, is involved in the rescue of stalled replication forks, and therefore prevents genomic instability (Shibata et al., 2005). A potential role in fitness represents a novel functionality for this protein.

PGN_1300 is a transcriptional regulator of the HcpR (Crp/Fnr) family involved in resistance to nitrosative stress. In the oral cavity nitrosative stress is particularly relevant due to the high intake of dietary nitrate. The protective mechanisms against nitrosative stress are poorly understood in *P. gingivalis*; however, HcpR is required for growth with nitrite and nitric oxide (Boutrin et al., 2012; Lewis et al., 2012). HcpR has also been shown to play a significant role in sustaining *P. gingivalis* viability within epithelial and endothelial cells (Lewis et al., 2012). The current study confirms and extends these findings to show the importance of HcpR in the survival of *P. gingivalis in vivo*. In addition to HcpR, PGN_0004, and PGN_0959 are induced under nitric oxide stress (Boutrin et al., 2012) and these genes were also negatively selected in both the mouse and TIGK infection models. PGN_0004 is annotated as a NAD^+^-dependent sirtuin deacetylase CobB, the activity of which can also impact gene transcription (Zhou et al., 2017). Similarly, PGN_0959 is also annotated as a transcriptional regulator. In addition, rubreythrin (PGN_0302), present in both screens, can confer resistance to both oxidative and nitrosative stress (Mydel et al., 2006).

PGN_0770, *rnZ*, is annotated as RNase Z, a zinc phosphodiesterase, which displays tRNA 3′-processing endonuclease activity, and is involved in tRNA maturation in organisms that do not contain a chromosomally encoded CCA determinant (Pellegrini et al., 2003). RNase Z is widely distributed among bacteria (Condon and Putzer, 2002). In *E. coli* RNase Z plays a significant role in mRNA decay (Perwez and Kushner, 2006), and controls the levels of 6S RNA, a stable sRNA, and an important transcription regulator that acts by binding to the sigma 70-containing holoenzyme of RNA polymerase (Chen et al., 2016).

### Genes important for epithelial colonization or abscess formation only

Supplementary Tables 5, 6 show genes that were identified in only one of the screens. In the abscess model there were 14 unique genes, many of which are annotated as transporter or efflux proteins, and as hypothetical. PGN_1721 was recently shown to encode a serine-palmitoyl transferase that is required for sphingolipid synthesis in *P. gingivalis* (Moye et al., 2016); therefore, sphingolipid synthesis is likely essential in the abscess environment. Sphingolipid synthesis strongly impacts the presentation of surface polysaccharides and gingipains, and contributes to resistance to oxidative stress (Moye et al., 2016). Hence sphingolipids may play multiple important roles in the survival of *P. gingivalis in vivo*.

In the epithelial cell colonization model, there were 59 unique genes. While many were annotated as hypothetical, a number of previously characterized genes were identified. These included *clpC*, consistent with our previous report demonstrating that ClpC and ClpXP are necessary for entry into gingival epithelial cells (Capestany et al., 2008). Also present were *mfa2* and *mfa5*, the two genes of the *mfa* operon not represent in the combined screen. Mfa2 is the anchor and length regulator for the Mfa1 structure (Hasegawa et al., 2009), and Mfa5 is a tip component which possesses a von Willebrand factor type A (VWA) domain that may be involved in adherence to host cells (Hasegawa et al., 2016). *ptk1* encodes a tyrosine kinase which is required for secretion of extracellular polysaccharide and for optimal community development with *S. gordonii* (Wright et al., 2014). Tyrosine kinase activity would therefore also appear to be essential for epithelial cell colonization. *gppX* encodes a hybrid TCS which can control the expression of around 100 genes in *P. gingivalis* (Hirano et al., 2013). Loss of GppX produces a phenotype deficient in monospecies biofilm formation (Hirano et al., 2013), and the current data also establish a role for GppX in epithelial colonization. GppX also possesses a TPR motif which may contribute to epithelial cell colonization.

## Conclusions

Using Tn-Seq with two models and stringent selection criteria we established that around a quarter of the genes of the host-adapted pathogen *P. gingivalis* contribute to fitness *in vivo*. The results provided functional verification that many previous identified virulence factors, including fimbriae, proteolytic enzymes, extracellular polysaccharides and membrane lipids, iron acquisition systems, and tetratricopeptide repeat proteins, all contribute to fitness of the organism. Additionally, the ability to withstand oxidative and nitrosative stresses, to tightly regulate surface molecule expression, and to conjugatively transfer DNA, contribute to fitness in our model systems. A number of newly identified fitness associated genes revealed novel aspects to arginine metabolism, along with genome and RNA stability, in the pathobiology of *P. gingivalis*.

## Author contributions

DM, JH, YW, and ZN conceived and performed the experiments, DM, JP, DY, DS, MW, and RL conceived overall plan, interpreted data, and wrote sections of the manuscript.

### Conflict of interest statement

The authors declare that the research was conducted in the absence of any commercial or financial relationships that could be construed as a potential conflict of interest.

## Supplementary material

Supplementary Figure 1 Examples of number of insertions and location within genes. **(A)** PGN_1753 (not negatively selected) and **(B)** PGN_1444 (negatively selected). Genes are shown with the number of reads within the gene (based on height of the peak) as well as the location in the gene. Data obtained using CLC Genomic Workbench V7.2.

Supplementary Table 1 Primer list.

Supplementary Table 2 Library summary information.

Supplementary Table 3 Contribution of *P. gingivalis* genes to fitness.

Supplementary Table 4 Genes important for fitness in both TIGK and abscess models.

Supplementary Table 5 Genes important for fitness only in the TIGK model.

Supplementary Table 6 Genes important for fitness only in the abscess model.
