# Supplementary figures and images for: Genes Contributing to Porphyromonas gingivalis Fitness in Abscess and Epithelial Cell Colonization Environments
Source: Front Cell Infect Microbiol. 2017 Aug 28;7:378. doi: 10.3389/fcimb.2017.00378 (PMC5581868; doi:10.3389/fcimb.2017.00378)

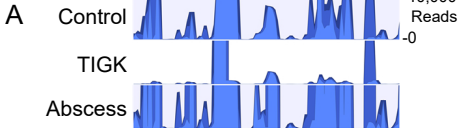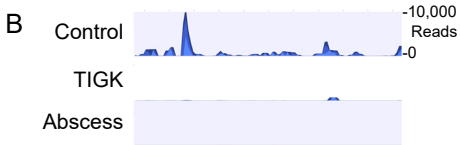

Supplement: Supplementary Figure 1 — Examples of number of insertions and location within genes. (A) PGN_1753 (not negatively selected) and (B) PGN_1444 (negatively selected). Genes are shown with the number of reads within the gene (based on height of the peak) as well as the location in the gene. Data obtained using CLC Genomic Workbench V7.2. [file Image1.pdf]
