# Supplementary material for: Genes Contributing to Porphyromonas gingivalis Fitness in Abscess and Epithelial Cell Colonization Environments
Source: Front Cell Infect Microbiol. 2017 Aug 28;7:378. doi: 10.3389/fcimb.2017.00378 (PMC5581868; doi:10.3389/fcimb.2017.00378)
Supplement: Supplementary Table 1 — Primer list. [file Table1.DOCX]

**Supplementary Table 1. Primer list**

| **Primer** | **5` -> 3` sequence** | **Use** |
| --- | --- | --- |
| Adapt_F_ctrl | TTCCCTACACGACGCTCTTCCGATCTTTTCCNN | Barcoded adaptors for Illumina sequencing |
| Adapt_R_ctrl | GGAAAAGATCGGAAGAGCGTCGTGTAGGGAA |  |
| Adapt_F_tigk | TTCCCTACACGACGCTCTTCCGATCTGGGAANN |  |
| Adapt_R_tigk | TTCCCAGATCGGAAGAGCGTCGTGTAGGGAA |  |
| Adapt_F_abscess | TTCCCTACACGACGCTCTTCCGATCTAAAGGNN |  |
| Adapt_R_abscess | CCTTTAGATCGGAAGAGCGTCGTGTAGGGAA |  |
| Lib_3 | CAAGCAGAAGACATACGAAGACCGGGGACTTATCATCCAACCTGT | PCR prior to sequencing |
| Lib_5 | AATGATACGGCGACCGAACACTCTTTCCCTACACGACGCTCTTCCGATCT |  |
| 16s rRNA F | AGG AAC TCC GAT TGC GAA GG | qRT-PCR |
| 16s rRNA R | TCG TTT ACT GCG TGG ACT ACC |  |
| ermF-F | GCTGATTTGACAGTTGGCGG |  |
| ermF-R | TCTGGGAGGTTCCATTGTCC |  |
| tetQ-F | AATTACTGTTCGGGCTTCTA |  |
| tetQ-R | GCTTGTATGCCTTCCTTTGC |  |
| PGN_0770_A_F | CGATGCTCCGAAAGAAAGCCATTCAGATC | Mutant strain construction |
| PGN_0770_erm_R | ACGGGCAATTTCTTTTTTGTCATATTGCACAAAGATATATAATGTG |  |
| PGN_0770_erm_F | GATGGAGCGGAAACGTAAAAGAACGCCTTTTCCGGCAG |  |
| PGN_0770_B_R | CGACGTATGCGGTTTATTCCCTCGTAGTATGC |  |
| PGN_1300_A_F | CTATGGACAATATATTGCAGGCG |  |
| PGN_1300_erm_R | ACGGGCAATTTCTTTTTTGTCATCGCAGCATCGTTTCCTTCAG |  |
| PGN_1300_erm_F | GATGGAGCGGAAACGTAAAAGATCAAATTAAATCTTCTTTAGTCGTCC |  |
| PGN_1300_B_R | CCTGTATTATAATGTATAGGAGGCC |  |
| PGN_1200_A_F | ACGGCCAGTGAATTCTATCCGCTTTGCGGCCC |  |
| PGN_1200_A_R | GAGATAATTCGTTGTGCCGCTAAGGTACATAATTGGGATAC |  |
| PGN_1200_tet_F | ACAACGAATTATCTCCTTAACGTAC |  |
| PGN_1200_tet_R | CAGAATGAAGAGTCATTTTATTGCCAAGTTCTAATGCTTCTATC |  |
| PGN_1200_B_F | TGACTCTTCATTCTGTACCGTCTC |  |
| PGN_1200_B_R | CCGGGTACCGAGCTCCTCGCTGAAGCACTTTTTCAG |  |
| PGN_1444_A_F | ACGGCCAGTGAATTCTATCTTGGAGCCTAAGAATTATCAGG |  |
| PGN_1444_A_R | GAGATAATTCGTTGTACGTTCTTAATACTCTTTCGATTCTG |  |
| PGN_1444_tet_F | ACAACGAATTATCTCCTTAACGTAC |  |
| PGN_1444_tet_R | CGTATGCTACTTTGCTTTTATTGCCAAGTTCTAATGCTTCTA |  |
| PGN_1444_B_F | GCAAAGTAGCATACGAATATGATAGAC |  |
| PGN_1444_B_R | CCGGGTACCGAGCTCCGTAACCCAACCCTAACTTG |  |
