# Supplementary material for: Genes Contributing to Porphyromonas gingivalis Fitness in Abscess and Epithelial Cell Colonization Environments
Source: Front Cell Infect Microbiol. 2017 Aug 28;7:378. doi: 10.3389/fcimb.2017.00378 (PMC5581868; doi:10.3389/fcimb.2017.00378)
Supplement: Supplementary Table 2 — Library summary information. [file Table2.DOCX]

**Supplementary Table 2: Library summary information**

| Library | Total Number of Reads | # of mapped reads (% total) | AVG # of reads/gene | RPKM^1^ |
| --- | --- | --- | --- | --- |
| Input | 23,692,415 | 6,483,184 (71) | 3010 | 413 |
| TIGK | 21,150,599 | 12,736,982 (85) | 5913 | 312 |
| Abscess | 29,256,415 | 11,568,835 (82) | 5370 | 688 |

^1^ Reads per kilobase of transcript per million reads mapped
