# Supplementary material for: Genes Contributing to Porphyromonas gingivalis Fitness in Abscess and Epithelial Cell Colonization Environments
Source: Front Cell Infect Microbiol. 2017 Aug 28;7:378. doi: 10.3389/fcimb.2017.00378 (PMC5581868; doi:10.3389/fcimb.2017.00378)
Supplement: Supplementary Table 4 — Genes important for fitness in both TIGK and abscess models. [file Table4.docx]

**Supplementary Table 4. Genes important for fitness in both TIGK and abscess models**^1^.

| **Feature ID** | **Log2 Fold change (TIGK)** | **Log2 Fold change (Abscess)** | **Gene description** | **Gene name** |
| --- | --- | --- | --- | --- |
| PGN_0002 | -7.285920016 | ND^2^ | conserved hypothetical protein |  |
| PGN_0003 | -7.386060785 | -7.967796882 | conserved hypothetical protein |  |
| PGN_0004 | -7.367877737 | -8.435036794 | putative nicotinate mononucleotide:5,6-dimethylbenzimidazole |  |
| PGN_0009 | -7.272891009 | -8.133599486 | glycosyl hydrolase family 3 |  |
| PGN_0010 | -3.901784046 | -4.854045532 | probable L-threonine-O-3-phosphate decarboxylase |  |
| PGN_0012 | ND | -6.970980161 | two-component system response regulator |  |
| PGN_0013 | -7.515132109 | -7.582292549 | putative two-component system sensor histidine kinase |  |
| PGN_0014 | -6.141882337 | -8.694479775 | conserved hypothetical protein |  |
| PGN_0016 | -7.562784287 | -8.842491777 | putative TIM-barrel protein |  |
| PGN_0017 | -6.103413711 | -5.150681521 | sulfate transporter permease |  |
| PGN_0020 | -5.622315435 | -5.086146843 | probable DNA-binding protein |  |
| PGN_0025 | -7.177359736 | -6.258462392 | probable SpoU rRNA methylase family protein |  |
| PGN_0030 | -7.577912163 | -8.645071149 | beta-mannosidase |  |
| PGN_0033 | -8.483352267 | -7.676048742 | thioredoxin |  |
| PGN_0039 | -6.656410561 | -5.308521062 | beta-hexosaminidase |  |
| PGN_0063 | -7.052220241 | -6.534419616 | conserved transmembrane protein found in conjugate transposon | traJ_1 |
| PGN_0064 | -7.34020618 | -4.893362211 | putative conserved protein found in conjugate transposon | traI_1 |
| PGN_0068 | -6.816356506 | -6.04701482 | hypothetical protein |  |
| PGN_0070 | -6.870192079 | -10.00774633 | hypothetical protein |  |
| PGN_0073 | -7.017999829 | -8.57058057 | putative conserved protein found in conjugate transposon | traA_2 |
| PGN_0075 | -4.769613076 | -6.662732454 | hypothetical protein |  |
| PGN_0076 | -5.89956236 | -3.992768431 | putative mobilization protein TraG family | traG |
| PGN_0079 | -7.772980914 | -3.362049012 | hypothetical protein |  |
| PGN_0080 | -8.159770445 | -6.226932336 | probable tetracycline resistance element mobilization regulatory protein RteC |  |
| PGN_0082 | -5.497005527 | -6.666032223 | probable transcriptional regulator AraC family |  |
| PGN_0084 | -6.359240026 | -5.193732325 | DNA topoisomerase I |  |
| PGN_0085 | -7.777518347 | -7.10771925 | hypothetical protein |  |
| PGN_0086 | -6.954173041 | -6.968862595 | putative DNA methylase |  |
| PGN_0087 | -7.013529256 | -9.955163116 | conserved hypothetical protein |  |
| PGN_0088 | -7.903351656 | -8.681006437 | transcriptional regulator | sinR |
| PGN_0090 | -7.159074103 | -8.695722447 | hypothetical protein |  |
| PGN_0094 | -6.428778891 | -8.343936299 | putative bacteriophage integrase |  |
| PGN_0109 | -7.814858002 | -8.882019377 | hypothetical protein |  |
| PGN_0110 | -7.456609046 | -10.52376678 | hypothetical protein |  |
| PGN_0120 | -7.492694423 | -7.744995249 | hypothetical protein |  |
| PGN_0121 | -6.940178499 | -7.91868438 | hypothetical protein |  |
| PGN_0122 | -3.8623522 | -7.464006553 | 28 kDa outer membrane protein Omp28 |  |
| PGN_0126 | -7.814050469 | -9.767040635 | putative transmembrane glucose/galactose transporter |  |
| PGN_0128 | -4.268209797 | -3.721810105 | immunoreactive 53 kDa antigen |  |
| PGN_0129 | -7.353614412 | -6.207775745 | hypothetical protein |  |
| PGN_0139 | -7.517354441 | -6.456510485 | hypothetical protein |  |
| PGN_0140 | -7.449173412 | -6.945385308 | peptide methionine sulfoxide reductase |  |
| PGN_0141 | -6.65515144 | -6.792699208 | conserved hypothetical protein |  |
| PGN_0147 | -6.465011258 | -7.11714437 | conserved hypothetical protein |  |
| PGN_0148 | -7.473786912 | -10.18140822 | conserved hypothetical protein |  |
| PGN_0149 | -5.0297179 | -5.189982654 | ribose-phosphate pyrophosphokinase |  |
| PGN_0152 | -6.367877737 | -6.435045131 | immunoreactive 61 kDa antigen |  |
| PGN_0155 | -7.33631017 | -8.555474916 | hypothetical protein |  |
| PGN_0156 | -7.502052081 | -4.392866916 | conserved hypothetical protein |  |
| PGN_0157 | ND | -5.117902789 | putative thiamine biosynthesis protein ThiH | thiH |
| PGN_0159 | -6.676831556 | -6.065916918 | probable thiamin-phosphate pyrophosphorylase |  |
| PGN_0160 | -6.238423848 | -3.524189078 | thiamine biosynthesis protein |  |
| PGN_0168 | -7.48475846 | -9.667393665 | lipopolysaccharide biosynthesis protein WbpB |  |
| PGN_0171 | ND | -6.923541422 | partial transposase in ISPg2 |  |
| PGN_0183 | -7.267573251 | -4.832231793 | Minor fimbrial component | fimC |
| PGN_0192 | -4.886745225 | -3.731509115 | conserved hypothetical protein |  |
| PGN_0196 | -5.20867332 | -9.083194749 | xanthine/uracil permease |  |
| PGN_0199 | -8.342532523 | -3.463230083 | hypothetical protein |  |
| PGN_0200 | -6.520956481 | -8.910046043 | conserved hypothetical protein |  |
| PGN_0221 | -5.442280035 | -7.509426657 | DNA topoisomerase III |  |
| PGN_0223 | -7.109141129 | -10.32314486 | glycosyl transferase family 4 |  |
| PGN_0224 | -7.516109726 | -10.16822959 | UDP-N-acetyl-D-mannosaminuronic acid dehydrogenase |  |
| PGN_0225 | -7.956823345 | -6.670344664 | probable glycosyltransferase |  |
| PGN_0226 | -3.893653439 | -5.808822633 | conserved hypothetical protein |  |
| PGN_0227 | ND | -4.864136609 | probable glycosyl transferase family 1 |  |
| PGN_0228 | -7.253658422 | -6.942303364 | probable coenzyme F390 synthetase |  |
| PGN_0229 | -7.004355318 | -5.684510197 | putative DNA-binding protein histone-like family |  |
| PGN_0230 | -7.988287039 | -6.248098349 | probable serine acetyltransferase |  |
| PGN_0231 | -7.345910822 | -6.219304069 | probable delta-aminolevulinic acid dehydratase |  |
| PGN_0232 | -4.607330314 | -5.951587795 | probable glycosyl transferase family 2 |  |
| PGN_0233 | -5.837842351 | -9.226929929 | conserved hypothetical protein with glycosyl transferase WecB/TagA/CpsF family |  |
| PGN_0237 | -7.315448488 | -7.449726433 | conserved hypothetical protein |  |
| PGN_0270 | -7.088533857 | -6.885610968 | probable amidophosphoribosyl-transferase |  |
| PGN_0271 | -7.063211699 | -5.400879436 | endopeptidase PepO |  |
| PGN_0277 | -6.089688814 | -9.156851635 | Heat shock protein 15 | hslR |
| PGN_0282 | -7.73755157 | -6.942221245 | 2',3'-cyclic-nucleotide 2'-phosphodiesterase precursor |  |
| PGN_0287 | -7.432884265 | -5.96790637 | Mfa fimbrillin | mfa1 |
| PGN_0289 | -7.156305596 | -7.692957175 | minor fimbrial component mfa3 | mfa3 |
| PGN_0290 | -7.780218221 | -10.11041545 | minor fimbrial component mfa4 | mfa4 |
| PGN_0295 | -3.444932049 | -7.915843535 | C-terminal domain of Arg- and Lys-gingipain proteinase |  |
| PGN_0302 | -7.269958853 | -8.551242987 | rubrerythrin | rbr |
| PGN_0303 | -6.018834436 | -3.610818156 | putative zinc protease |  |
| PGN_0306 | -8.827307768 | -5.139592279 | conserved hypothetical protein |  |
| PGN_0314 | -7.547557793 | -4.982126557 | probable formate/nitrite transporter |  |
| PGN_0316 | -7.39630508 | -8.24107269 | precorrin-4 C11-methyltransferase |  |
| PGN_0322 | -5.928228179 | -7.509957061 | conserved hypothetical protein |  |
| PGN_0329 | -6.69981534 | -5.030027022 | conserved hypothetical protein |  |
| PGN_0334 | -7.526757434 | -8.593913947 | conserved hypothetical protein |  |
| PGN_0344 | -6.390874006 | -5.949021261 | probable haloacid dehalogenase-like hydrolase |  |
| PGN_0345 | -7.31953665 | -4.468583317 | conserved hypothetical protein |  |
| PGN_0348 | -4.784451635 | -7.034479857 | putative universal stress protein UspA | uspA |
| PGN_0349 | -7.181370943 | -8.663568778 | upregulated in stationary phase protein A | ustA |
| PGN_0351 | -7.423569768 | -4.163498732 | pyruvate carboxylase subunit B |  |
| PGN_0361 | -7.302456148 | -7.84325528 | putative glycosyl transferase family 2 |  |
| PGN_0362 | -7.20867332 | ND | hypothetical protein |  |
| PGN_0373 | -7.492229751 | -10.23746214 | putative thioredoxin |  |
| PGN_0375 | -7.485145023 | -5.267385978 | phosphoribulose/uridine kinase |  |
| PGN_0388 | -7.623332832 | -10.38941516 | putative thiol peroxidase |  |
| PGN_0390 | -7.516897645 | -8.079586116 | conserved hypothetical protein |  |
| PGN_0391 | -7.520956481 | -8.543723993 | conserved hypothetical protein |  |
| PGN_0400 | -5.276533886 | -6.513601281 | conserved hypothetical protein |  |
| PGN_0405 | -6.631293485 | -4.827412574 | alpha-1,2-mannosidase family protein |  |
| PGN_0407 | -7.129015063 | -5.570857554 | hypothetical protein |  |
| PGN_0410 | -6.357076893 | -6.102301192 | probable RNA pseudouridylate synthase |  |
| PGN_0412 | -7.600998173 | -9.668156363 | Nucleoside triphosphate pyrophosphohydrolase | mazG |
| PGN_0416 | -7.918600905 | -9.092675227 | putative type II DNA modification methyltransferase |  |
| PGN_0417 | -8.163906244 | -9.816031642 | conserved hypothetical protein |  |
| PGN_0421 | -3.835014526 | -8.60256897 | conserved hypothetical protein |  |
| PGN_0426 | -6.466692369 | ND | conserved hypothetical protein |  |
| PGN_0429 | -6.357076893 | -4.102322191 | putative 4-alpha-glucanotransferase |  |
| PGN_0431 | -7.269874722 | -8.922001558 | conserved hypothetical protein |  |
| PGN_0444 | -7.252097703 | ND | probable outer membrane efflux protein |  |
| PGN_0446 | ND | -6.820153428 | putative ABC transporter ATP-binding protein |  |
| PGN_0447 | -7.487944515 | -10.72503388 | putative ABC transporter permease protein |  |
| PGN_0448 | -6.462575888 | -9.404204897 | putative ABC transporter ATP-binding protein |  |
| PGN_0450 | -6.950445084 | -4.185945812 | putative RNA polymerase sigma-70 factor ECF subfamily |  |
| PGN_0458 | -7.82273652 | -6.667508983 | hypothetical protein |  |
| PGN_0460 | -7.698065642 | -7.511468039 | DNA-binding protein histone-like family |  |
| PGN_0462 | -7.437926808 | -8.282694626 | probable haloacid dehalogenase-like hydrolase |  |
| PGN_0463 | -7.957287809 | -9.975538479 | conserved hypothetical protein |  |
| PGN_0464 | -7.456116171 | -9.119921565 | conserved hypothetical protein |  |
| PGN_0471 | -4.244734868 | -3.90303827 | conserved hypothetical protein |  |
| PGN_0481 | -7.69149229 | -5.521616142 | hypothetical protein |  |
| PGN_0483 | -6.766674661 | -6.959364456 | putative aldose 1-epimerase |  |
| PGN_0484 | -7.747494819 | -4.814652957 | putative galactokinase |  |
| PGN_0485 | -7.88675132 | -9.953911234 | conserved hypothetical protein |  |
| PGN_0488 | -6.415522073 | -7.89773052 | conserved hypothetical protein |  |
| PGN_0491 | -7.515416002 | -5.930500811 | phosphotyrosine protein phosphatase ltp1 |  |
| PGN_0492 | -5.778155999 | -5.663088396 | cation-transporting ATPase |  |
| PGN_0508 | -7.246389084 | -7.176043463 | putative aminopeptidase |  |
| PGN_0513 | -7.043257003 | -8.695377948 | conserved hypothetical protein |  |
| PGN_0530 | -6.619192892 | -8.101397952 | conserved hypothetical protein |  |
| PGN_0531 | -7.451532591 | -8.518696426 | putative von Willebrand factor type A |  |
| PGN_0532 | -6.453566963 | ND | magnesium chelatase subunit I |  |
| PGN_0533 | -7.452208288 | -8.178336193 | putative quinolinate synthetase complex subunit A |  |
| PGN_0534 | -8.062008349 | -7.866135252 | putative nicotinate-nucleotide pyrophosphorylase |  |
| PGN_0535 | -7.15579982 | -3.385292974 | L-aspartate oxidase |  |
| PGN_0539 | -7.419505183 | -7.050566996 | metallo-beta-lactamase superfamily protein |  |
| PGN_0556 | -7.292689738 | -8.22554487 | putative cobalamin biosynthesis-related protein | hmuS |
| PGN_0557 | -7.376229598 | -7.788881432 | TonB-dependent receptor | hmuR |
| PGN_0558 | -6.931139344 | -7.998302708 | conserved hypothetical protein | hmuY |
| PGN_0559 | ND | ND | conserved hypothetical protein |  |
| PGN_0561 | -7.249976204 | -6.791670836 | trypsin like proteinase PrtT | prtT |
| PGN_0563 | -7.11455443 | -11.42964421 | conserved hypothetical protein |  |
| PGN_0564 | -7.633453008 | ND | superoxide dismutase Fe-Mn | sod |
| PGN_0566 | -7.350674037 | -10.50529848 | probable thioesterase protein |  |
| PGN_0580 | -5.834281417 | -5.316471616 | conserved hypothetical protein |  |
| PGN_0581 | -9.61920023 | -7.364432358 | conserved hypothetical protein |  |
| PGN_0582 | -6.516109726 | -3.828428485 | DNA topoisomerase I |  |
| PGN_0588 | ND | -6.768118145 | conserved hypothetical protein |  |
| PGN_0590 | -7.625781889 | -7.10798073 | putative Fic family protein |  |
| PGN_0591 | -7.486666111 | -5.909965067 | conserved hypothetical protein |  |
| PGN_0592 | -7.468371524 | -10.12049191 | putative conserved protein found in conjugate transposon | traQ_1 |
| PGN_0593 | -7.574805422 | -7.057006131 | putative conserved protein found in conjugate transposon | traO_1 |
| PGN_0594 | -8.69410363 | -9.761265324 | conserved protein found in conjugate transposon | traN_2 |
| PGN_0596 | -7.481694505 | ND | conserved hypothetical protein found in conjugate transposon |  |
| PGN_0597 | -6.19053585 | -8.257689551 | putative conserved protein found in conjugate transposon | traK_2 |
| PGN_0598 | -7.995275336 | -6.062445556 | conserved transmembrane protein found in conjugate transposon | traJ_2 |
| PGN_0604 | -7.437036268 | -11.56308947 | ferritin |  |
| PGN_0606 | -6.669862991 | -5.929672662 | glucosamine-6-phosphate isomerase |  |
| PGN_0607 | -5.940824507 | -7.85598347 | dipeptidyl peptidase 11 |  |
| PGN_0613 | -7.497412776 | -8.259719729 | UDP-glucose 6-dehydrogenase |  |
| PGN_0637 | -7.494671612 | -9.09686798 | putative heat shock-related protease htrA protein |  |
| PGN_0641 | -6.72310799 | -4.49971835 | 50S ribosomal protein L9 | rplI |
| PGN_0643 | -8.220649185 | -9.287811634 | 3,4-dihydroxy-2-butanone 4-phosphate synthase |  |
| PGN_0648 | -5.45338588 | -7.172617522 | conserved hypothetical protein |  |
| PGN_0649 | -6.828377707 | -8.264775672 | conserved hypothetical protein |  |
| PGN_0650 | -7.284504879 | -6.133830636 | conserved hypothetical protein |  |
| PGN_0653 | -7.043257003 | ND | conserved hypothetical protein |  |
| PGN_0654 | -8.386595423 | -6.753310363 | conserved hypothetical protein |  |
| PGN_0656 | -6.906650126 | -6.973806407 | conserved hypothetical protein |  |
| PGN_0658 | -6.366479591 | -10.3722317 | GTP-binding elongation factor family protein TypA/BipA |  |
| PGN_0661 | -5.615357439 | -3.405720127 | alkyl hydroperoxide reductase F subunit |  |
| PGN_0663 | ND | ND | conserved hypothetical protein |  |
| PGN_0668 | -7.20867332 | ND | RNA-binding protein |  |
| PGN_0672 | -7.329410671 | -6.452661322 | probable biopolymer transport protein |  |
| PGN_0673 | -7.494287593 | -9.017125757 | probable biopolymer transport protein |  |
| PGN_0674 | -8.002224318 | -9.391316635 | conserved hypothetical protein |  |
| PGN_0675 | -6.99614014 | -7.670989004 | conserved hypothetical protein |  |
| PGN_0688 | -6.835759893 | -7.487888257 | conserved hypothetical protein |  |
| PGN_0700 | -7.453566963 | -6.672722337 | putative oxidoreductase Gfo/Idh/MocA family |  |
| PGN_0701 | -3.528071165 | -6.33933339 | Beta-galacosidase | lacZI |
| PGN_0703 | -7.414786697 | -4.2340414 | hypothetical protein |  |
| PGN_0704 | -7.630907776 | -4.125155131 | putative tonB-linked outer membrane receptor |  |
| PGN_0707 | -6.900287199 | -8.659321645 | putative iron compound ABC transporter permease protein |  |
| PGN_0709 | -7.467116418 | -6.133912814 | putative indolepyruvate ferredoxin oxidoreductase beta subunit |  |
| PGN_0711 | -7.339609534 | -10.72869774 | putative oxidoreductase short chain dehydrogenase/reductase family |  |
| PGN_0714 | -8.689805832 | -9.756964889 | probable pyrazinamidase/nicotinamidase |  |
| PGN_0715 | -8.128334649 | -4.930784638 | outer membrane efflux protein |  |
| PGN_0716 | -6.49818703 | -6.211713117 | ABC transporter permease |  |
| PGN_0718 | -7.262498931 | -8.011696894 | putative ABC transporter permease protein |  |
| PGN_0719 | -7.688669941 | -5.242221395 | probable ABC transporter permease protein |  |
| PGN_0720 | -7.812748493 | -6.616872265 | putative ABC transporter permease protein |  |
| PGN_0736 | ND | -9.548856579 | putative nicotinate-nucleotide-dimethylbenzimidazole phosphoribosyltransferase |  |
| PGN_0738 | -7.232526457 | ND | putative phosphoglycerate mutase |  |
| PGN_0740 | ND | -8.174156706 | conserved hypothetical protein |  |
| PGN_0741 | -7.451532591 | -8.11942362 | TonB-dependent receptor |  |
| PGN_0750 | -8.102689622 | -5.040585628 | putative copper homeostasis protein CutC |  |
| PGN_0754 | -7.515920562 | -9.101550282 | conserved hypothetical protein |  |
| PGN_0758 | -8.461659517 | ND | conserved hypothetical protein |  |
| PGN_0759 | -8.715416373 | -5.612647378 | conserved hypothetical protein |  |
| PGN_0770 | -9.308814541 | ND | conserved hypothetical protein |  |
| PGN_0771 | -7.675152568 | -11.12082689 | conserved hypothetical protein |  |
| PGN_0774 | -6.163317579 | -7.23048164 | two-component system sensor histidine kinase |  |
| PGN_0780 | -7.908008251 | -5.402278769 | PrtQ, protease | prtQ |
| PGN_0782 | -7.255982181 | -8.738183481 | putative tRNA pseudouridine synthase A | truA |
| PGN_0784 | -7.367877737 | -6.265118046 | hypothetical protein |  |
| PGN_0786 | -8.967122485 | -7.226932336 | conserved hypothetical protein |  |
| PGN_0788 | -8.002741895 | -10.23982643 | peptidyl-dipeptidase |  |
| PGN_0793 | -6.844046634 | -4.434361346 | 4-alpha-glucanotransferase |  |
| PGN_0795 | -6.813537515 | -8.073343611 | conserved hypothetical protein |  |
| PGN_0807 | -4.127880861 | -11.4865917 | putative DNAse related protein |  |
| PGN_0808 | -5.729824644 | -9.302874214 | probable isoprenyl synthetase |  |
| PGN_0811 | -6.922792504 | -3.854793598 | conserved hypothetical protein |  |
| PGN_0812 | -6.272807049 | -5.551485256 | conserved hypothetical protein |  |
| PGN_0826 | -7.741311922 | -9.808473543 | dihydrolipoamide dehydrogenase |  |
| PGN_0827 | -7.34618571 | -9.528819378 | glucosamine-6-phosphate isomerase | nagB |
| PGN_0836 | -5.840941535 | -7.270678437 | conserved hypothetical protein |  |
| PGN_0852 | -7.276664151 | -8.20008939 | immunoreactive 47 kDa antigen |  |
| PGN_0857 | -7.224310445 | -5.862624961 | conserved hypothetical protein |  |
| PGN_0862 | -6.864879462 | -7.195071948 | Type III restriction enzyme, res subunit |  |
| PGN_0863 | -5.951401292 | -9.018564632 | DNA methylase N-4/N-6 |  |
| PGN_0872 | -7.525325043 | -7.592479462 | DNA-binding protein histone-like family |  |
| PGN_0874 | -7.141565626 | -9.083194749 | conserved hypothetical protein |  |
| PGN_0876 | -7.305003781 | -9.857592286 | TPR domain protein |  |
| PGN_0880 | -7.032431496 | -4.081680489 | tyrosine phenol-lyase | tnaA |
| PGN_0898 | -7.20639903 | -7.236722207 | probable peptidylarginine deiminase |  |
| PGN_0900 | -7.773976551 | -9.815144695 | thiol protease |  |
| PGN_0903 | -6.984281731 | ND | two-component system response regulator FimR | fimR |
| PGN_0904 | -7.063848043 | -7.831452931 | two-component system sensor histidine kinase FimS | fimS |
| PGN_0917 | -7.140993327 | -6.902338934 | tyrosine type site-specific recombinase |  |
| PGN_0922 | -7.103980136 | -7.908104353 | conserved hypothetical protein |  |
| PGN_0926 | -5.892755297 | -5.822398749 | conserved hypothetical protein |  |
| PGN_0929 | -6.888110029 | -7.955277926 | conserved hypothetical protein |  |
| PGN_0930 | -7.24899016 | -9.053111336 | hypothetical protein |  |
| PGN_0933 | -7.30178882 | -3.845389686 | probable transcriptional regulator |  |
| PGN_0959 | -6.359240026 | -6.748327048 | probable transcriptional regulator |  |
| PGN_0960 | -7.916494559 | -6.813729894 | conserved hypothetical protein |  |
| PGN_0980 | -6.805730976 | -5.317376433 | putative alpha-1,2-mannosidase family protein |  |
| PGN_0991 | -6.892743156 | -9.281837321 | putative ribonuclease BN |  |
| PGN_1009 | -6.469039382 | -7.355624191 | calcium-transporting ATPase |  |
| PGN_1011 | -4.224657734 | -5.761258675 | xanthine/uracil/vitamin C permease |  |
| PGN_1016 | -6.957798547 | -9.140438695 | conserved hypothetical protein |  |
| PGN_1035 | -7.568898556 | -8.909077025 | conserved hypothetical protein with DUF1063 domain |  |
| PGN_1036 | -7.281930027 | ND | putative G/U mismatch-specific DNA glycosylase |  |
| PGN_1037 | -6.840224024 | -8.122403534 | conserved hypothetical protein |  |
| PGN_1038 | -6.505652789 | -6.679733429 | conserved hypothetical protein |  |
| PGN_1039 | -7.552292526 | -4.767601835 | putative alpha-1,2-mannosidase precursor |  |
| PGN_1040 | -6.474387369 | -9.126505734 | conserved hypothetical protein |  |
| PGN_1041 | -7.465019424 | -7.309785551 | cytochrome d ubiquinol oxidase subunit I |  |
| PGN_1042 | -7.770412824 | -6.115116499 | cytochrome d ubiquinol oxidase subunit II |  |
| PGN_1043 | -8.096203862 | -7.163367902 | conserved hypothetical protein |  |
| PGN_1045 | -6.79516951 | -7.254641281 | Beta-galacosidase | lacZII |
| PGN_1049 | -7.194697767 | -6.928666412 | putative alkaline phosphatase |  |
| PGN_1050 | -7.632704448 | -8.114903146 | conserved hypothetical protein |  |
| PGN_1052 | -8.483743238 | -10.05340463 | conserved hypothetical protein |  |
| PGN_1061 | -7.454068938 | -9.52123334 | conserved hypothetical protein |  |
| PGN_1071 | -6.391183431 | -8.59584302 | hypothetical protein |  |
| PGN_1073 | -7.002229945 | -7.069390689 | hypothetical protein |  |
| PGN_1081 | -8.337960795 | -6.083192089 | conserved hypothetical protein |  |
| PGN_1092 | -9.092950016 | ND | ATP-dependent exoDNAse alpha subunit |  |
| PGN_1093 | -7.179530955 | -7.568617397 | conserved hypothetical protein |  |
| PGN_1094 | -6.850311876 | -7.244563634 | glycine dehydrogenase |  |
| PGN_1100 | -6.856375726 | -5.923529538 | putative capsule biosynthesis protein CapA |  |
| PGN_1103 | -6.809581651 | -9.876742542 | dipeptidase |  |
| PGN_1106 | -6.716826319 | -8.783986959 | conserved hypothetical protein |  |
| PGN_1107 | -7.766674661 | ND | conserved hypothetical protein |  |
| PGN_1109 | -8.466692369 | -3.626906331 | hypothetical protein |  |
| PGN_1111 | -5.920174182 | -5.739416093 | formate-tetrahydrofolate ligase |  |
| PGN_1112 | -5.832940634 | -3.950095094 | PhoH-like protein |  |
| PGN_1116 | -3.38294387 | -3.794103899 | putative aminotransferase |  |
| PGN_1117 | -3.794519841 | -8.044907715 | acetyl-CoA synthetase |  |
| PGN_1120 | -5.496302553 | -3.512353322 | putative NADPH-NAD transhydrogenase |  |
| PGN_1121 | -8.083149531 | -7.150305802 | probable NADPH-NAD transhydrogenase alpha subunit |  |
| PGN_1122 | -7.390039946 | -6.373787394 | NADPH-NAD transhydrogenase beta subunit |  |
| PGN_1126 | -8.748870422 | -4.686724618 | putative error-prone repair: SOS-response transcriptional repressor UmuD homolog |  |
| PGN_1127 | -7.882447416 | -6.547511524 | putative SOS mutagenesis and repair protein UmuC homolog |  |
| PGN_1128 | -7.744732946 | -8.226927521 | L-lactate permease |  |
| PGN_1141 | -8.238423848 | -7.305587551 | putative amidohydrolase |  |
| PGN_1146 | -8.056675645 | ND | conserved hypothetical protein |  |
| PGN_1149 | -6.659339486 | -9.726500879 | prolyl tripeptidase A |  |
| PGN_1158 | -7.628576771 | -4.714410388 | putative purine nucleoside phosphorylase |  |
| PGN_1159 | -7.434428071 | -5.290424404 | conserved hypothetical protein |  |
| PGN_1174 | -7.991782585 | -6.358501759 | electron transfer flavoprotein alpha subunit |  |
| PGN_1175 | -6.728056551 | -6.695673735 | putative enoyl-CoA hydratase |  |
| PGN_1176 | -7.286677885 | -5.546493227 | putative 3-hydroxybutyryl-CoA dehydrogenase |  |
| PGN_1180 | -7.559178216 | ND | conserved hypothetical protein |  |
| PGN_1197 | -6.285587168 | -9.160110925 | conserved hypothetical protein |  |
| PGN_1198 | -7.155617697 | -7.807741306 | putative sodium-solute transporter |  |
| PGN_1199 | -7.435186851 | -4.966292024 | DNA-binding protein histone-like family |  |
| PGN_1200 | -9.225115834 | -5.832839392 | ATPase AAA family |  |
| PGN_1201 | -5.800485793 | -7.505081091 | conserved hypothetical protein |  |
| PGN_1210 | -6.424183089 | -10.07630789 | hypothetical protein |  |
| PGN_1211 | -7.451532591 | -9.103657616 | hypothetical protein |  |
| PGN_1213 | -7.951045703 | -9.696277301 | putative ATP-binding protein |  |
| PGN_1214 | -7.957618649 | -9.60974019 | hypothetical protein |  |
| PGN_1227 | -7.161152043 | -6.22831847 | TPR domain protein |  |
| PGN_1230 | -5.582556003 | -4.102238194 | conserved hypothetical protein |  |
| PGN_1243 | -6.809581651 | -7.461708623 | UDP-glucose 6-dehydrogenase |  |
| PGN_1245 | -6.844385653 | -6.622037173 | long-chain-fatty-acid-CoA ligase |  |
| PGN_1246 | -7.125547789 | -5.813345111 | hypothetical protein |  |
| PGN_1247 | -7.056670226 | -7.386871287 | hypothetical protein |  |
| PGN_1252 | -7.036492344 | ND | iron-containing alcohol dehydrogenase |  |
| PGN_1253 | -6.798970704 | -8.451095692 | hypothetical protein |  |
| PGN_1268 | -7.30178882 | -7.182245358 | putative oxidoreductase |  |
| PGN_1269 | -7.123293723 | -6.39386234 | putative electron transport protein |  |
| PGN_1270 | -7.54611504 | -8.512187373 | conserved hypothetical protein |  |
| PGN_1271 | -5.960001932 | -9.027159582 | probable thioesterase superfamily protein |  |
| PGN_1300 | -7.874169892 | -8.52629578 | probable transcriptional regulator |  |
| PGN_1306 | -8.689805832 | -9.756964889 | hypothetical protein |  |
| PGN_1308 | -8.995275336 | -4.933147698 | probable iron dependent repressor |  |
| PGN_1309 | -6.350037492 | -8.334733623 | ferrous iron transport protein B | feoB |
| PGN_1310 | -6.842501206 | -6.839266787 | glycogen synthase |  |
| PGN_1313 | -7.27294698 | -5.804053559 | conserved hypothetical protein |  |
| PGN_1317 | -6.143087246 | -7.380175312 | conserved hypothetical protein |  |
| PGN_1321 | -5.458906907 | -10.98549687 | conserved hypothetical protein |  |
| PGN_1323 | -7.565254434 | -9.28555018 | TPR domain protein |  |
| PGN_1329 | -7.639087423 | -9.776640292 | hypothetical protein |  |
| PGN_1332 | ND | -9.150308341 | putative para-aminobenzoate synthase component I |  |
| PGN_1334 | -7.423494135 | -3.630987837 | conserved hypothetical protein |  |
| PGN_1335 | -6.932262004 | -6.699870851 | conserved hypothetical protein |  |
| PGN_1336 | -5.375039431 | -3.897918043 | conserved hypothetical protein |  |
| PGN_1338 | -4.733408576 | -7.994936055 | pyruvate phosphate dikinase |  |
| PGN_1339 | -6.993210143 | -10.07195837 | conserved hypothetical protein |  |
| PGN_1340 | -6.680296223 | -6.035975744 | conserved hypothetical protein |  |
| PGN_1347 | -6.41482897 | -4.136109338 | putative TonB-dependent receptor exported protein |  |
| PGN_1348 | -7.87669497 | -4.598067392 | conserved hypothetical protein |  |
| PGN_1349 | -7.031516504 | -3.617533583 | probable dipeptidyl aminopeptidase |  |
| PGN_1361 | -7.698392136 | -5.765561266 | conserved hypothetical protein |  |
| PGN_1362 | -4.202652797 | -3.984407065 | probable exported transglycosylase protein |  |
| PGN_1363 | -7.115563915 | -7.597769518 | ABC transporter ATP-binding protein |  |
| PGN_1366 | -6.920102707 | -5.40653667 | conserved hypothetical protein |  |
| PGN_1369 | -7.605427662 | -5.636421614 | conserved hypothetical protein |  |
| PGN_1370 | -4.430619811 | -5.50417544 | NAD-dependent nucleotide-diphosphate-sugar epimerase |  |
| PGN_1374 | -8.117705431 | -9.769827928 | putative ribosomal large subunit pseudouridine synthase D |  |
| PGN_1382 | -5.587184943 | ND | putative 3-dehydroquinate synthase |  |
| PGN_1402 | -7.029706859 | ND | putative amidinotransferase |  |
| PGN_1403 | -6.838359344 | -6.320556883 | ornithine aminotransferase |  |
| PGN_1404 | ND | -9.795213352 | conserved hypothetical protein |  |
| PGN_1409 | -6.172147444 | ND | putative peptidase |  |
| PGN_1411 | -5.603537333 | -7.840620587 | putative N-ethylammeline chlorohydrolase |  |
| PGN_1412 | -5.166313748 | -9.541613569 | putative purine nucleoside phosphorylase I |  |
| PGN_1416 | -7.003680935 | -7.402219069 | probable lysyl endopeptidase precursor |  |
| PGN_1422 | -6.859783238 | -7.774938568 | putative partial DNA-binding protein histone-like family |  |
| PGN_1424 | -8.591806557 | -7.658968342 | putative partial Type II restriction enzyme |  |
| PGN_1425 | -7.816503711 | -7.491348065 | putative partial Type II restriction enzyme |  |
| PGN_1426 | -7.285920016 | -8.353080645 | adenine-specific methyltransferase |  |
| PGN_1427 | -7.314778083 | -11.62986794 | hypothetical protein |  |
| PGN_1436 | -4.782932705 | -7.435036794 | conserved hypothetical protein |  |
| PGN_1443 | -7.521286349 | -4.480975953 | carbamoyl-phosphate synthase large subunit |  |
| PGN_1444 | -5.590871444 | -12.14989824 | carbamoyl-phosphate synthase small subunit |  |
| PGN_1445 | -7.514879713 | -6.453204775 | putative amidophosphoribosyltransferase |  |
| PGN_1446 | -5.278542382 | -8.03022019 | conserved hypothetical protein |  |
| PGN_1450 | -7.33459509 | -4.600150793 | putative tRNA isopentenyltransferase | miaA_1 |
| PGN_1463 | -6.936602607 | -10.00375963 | probable UbiA prenyltransferase |  |
| PGN_1464 | -5.606738317 | -5.306189316 | probable HAD-superfamily subfamily IB hydrolase |  |
| PGN_1466 | -7.20121236 | -3.415082583 | arginine-specific cysteine proteinase | rgpB |
| PGN_1467 | -7.611644543 | -10.26376566 | conserved hypothetical protein |  |
| PGN_1469 | -6.865683789 | -6.871449416 | dipeptidyl peptidase IV | dppIV |
| PGN_1474 | -7.912811512 | -7.732045288 | putative autoinducer-2 production protein LuxS |  |
| PGN_1475 | -7.696967526 | ND | probable 5'-methylthioadenosine/S-adenosylhomocysteine nucleosidase |  |
| PGN_1476 | -7.884872612 | -6.559721231 | conserved hypothetical protein |  |
| PGN_1479 | -7.421871272 | -8.142857574 | dipeptidyl peptidase 7 |  |
| PGN_1480 | -5.571252069 | ND | conserved hypothetical protein with DUF389 domain |  |
| PGN_1494 | -7.844046634 | -7.209287641 | putative oxygen-independent coproporphyrinogen III oxidase |  |
| PGN_1498 | -6.403318857 | -6.392489162 | conserved hypothetical protein |  |
| PGN_1505 | -7.527398809 | -7.009593513 | putative folylpolyglutamate synthase |  |
| PGN_1506 | -7.156993166 | -8.04684571 | putative transporter |  |
| PGN_1519 | -5.728056551 | -7.602565259 | hemagglutinin-related protein |  |
| PGN_1520 | -8.425908231 | -6.685715936 | conserved hypothetical protein |  |
| PGN_1523 | -5.431054594 | -3.425862086 | putative polysaccharide export outer membrane protein |  |
| PGN_1534 | -5.945654519 | -7.012803293 | hypothetical protein |  |
| PGN_1535 | -7.402449327 | -8.1476832 | hypothetical protein |  |
| PGN_1536 | -7.61483497 | -7.213220895 | hypothetical protein |  |
| PGN_1537 | -7.323117828 | -5.308484658 | probable cation efflux system protein |  |
| PGN_1538 | -7.664745926 | -4.788476912 | putative cation efflux system |  |
| PGN_1540 | -7.288847622 | -7.397828626 | putative ABC transport membrane protein |  |
| PGN_1548 | -6.842501206 | -5.398692395 | conserved hypothetical protein |  |
| PGN_1556 | -7.642752106 | -4.358255587 | putative hemagglutinin |  |
| PGN_1581 | -7.114762629 | -4.001982343 | DNA mismatch repair protein MutS |  |
| PGN_1591 | -7.832516637 | -5.961090785 | conserved hypothetical protein |  |
| PGN_1594 | -7.196607044 | -5.356883284 | DNA topoisomerase IV B subunit |  |
| PGN_1611 | -8.668108485 | -7.413340207 | conserved hypothetical protein |  |
| PGN_1617 | -7.582556003 | -6.327795131 | probable metallophosphoesterase |  |
| PGN_1618 | -7.581012228 | -10.35866548 | methionine gamma-lyase |  |
| PGN_1621 | -6.403318857 | ND | conserved hypothetical protein |  |
| PGN_1623 | -6.425912426 | -9.171141762 | conserved hypothetical protein |  |
| PGN_1632 | -4.664369059 | -5.079420781 | hypothetical protein |  |
| PGN_1637 | -6.840941535 | -6.685715936 | putative methenyltetrahydrofolate cyclohydrolase |  |
| PGN_1639 | -7.491885156 | -7.764984349 | conserved hypothetical protein |  |
| PGN_1641 | -6.104378598 | -8.097547593 | arginine/ornithine transport system ATPase |  |
| PGN_1646 | -7.780992435 | -9.333578032 | seryl-tRNA synthetase |  |
| PGN_1666 | -6.420735023 | -5.902917719 | phosphoribosylformylglycinamidine synthase |  |
| PGN_1670 | -6.867081012 | -7.555731811 | conserved hypothetical protein with predicted lysozyme domain |  |
| PGN_1671 | -7.006219468 | -5.741709662 | probable chromate transport protein |  |
| PGN_1682 | -7.65871134 | -7.363302512 | probable ABC transporter permease protein |  |
| PGN_1685 | -7.142985174 | -8.480236843 | NADP-dependent malate dehydrogenase |  |
| PGN_1691 | -7.516393427 | -7.415767116 | putative cysteine desulfurase |  |
| PGN_1692 | -7.675089038 | -10.64913939 | conserved hypothetical protein |  |
| PGN_1694 | -6.712485731 | -7.024164514 | putative alanyl dipeptidyl peptidase |  |
| PGN_1703 | -8.214678535 | ND | putative ribonuclease III |  |
| PGN_1714 | -4.28132733 | -5.278133471 | transcription-repair coupling factor |  |
| PGN_1720 | -8.196607044 | ND | hypothetical protein |  |
| PGN_1723 | -7.604657003 | -10.67181613 | conserved hypothetical protein |  |
| PGN_1724 | -7.571691977 | -6.638855809 | conserved hypothetical protein |  |
| PGN_1730 | -7.29896984 | -8.366129842 | conserved hypothetical protein |  |
| PGN_1733 | -3.443606651 | -5.574858391 | hemagglutinin protein | hagA |
| PGN_1735 | -7.277194395 | -4.975125723 | conserved hypothetical protein |  |
| PGN_1741 | -6.246256057 | -9.898379185 | conserved hypothetical protein |  |
| PGN_1744 | -8.196607044 | ND | conserved hypothetical protein |  |
| PGN_1745 | -7.557287542 | -8.946377043 | putative NapC/NirT cytochrome c-type protein |  |
| PGN_1746 | -7.650513272 | -8.717676423 | cytochrome c nitrite reductase catalytic subunit NrfA |  |
| PGN_1747 | -6.659339486 | -6.141534973 | conserved hypothetical protein |  |
| PGN_1748 | -8.552084986 | -5.159830981 | putative cytochrome c biogenesis protein CcsA |  |
| PGN_1769 | -7.705867346 | -9.18806211 | hypothetical protein |  |
| PGN_1770 | -5.122217146 | -6.416130378 | conserved hypothetical protein |  |
| PGN_1777 | -7.45228243 | -4.54293899 | bleomycin hydrolase |  |
| PGN_1779 | -6.056670226 | ND | conserved hypothetical protein |  |
| PGN_1780 | -7.258622563 | -9.69775471 | putative YjgF-like protein |  |
| PGN_1787 | -7.420726603 | -8.487888257 | probable 5-formyltetrahydrofolate cyclo-ligase |  |
| PGN_1792 | ND | -4.876762491 | glycerate kinase |  |
| PGN_1793 | -9.530605316 | -5.739794109 | conserved hypothetical protein |  |
| PGN_1795 | -8.08096139 | -7.411155037 | conserved hypothetical protein |  |
| PGN_1799 | ND | -7.860801441 | hypothetical protein |  |
| PGN_1808 | -7.672474845 | -8.739635489 | conserved hypothetical protein |  |
| PGN_1811 | -8.426864355 | -7.318181243 | putative alpha-L-fucosidase |  |
| PGN_1814 | -9.179839441 | -5.574585959 | conserved hypothetical protein |  |
| PGN_1816 | -6.984783003 | -8.730014924 | conserved hypothetical protein |  |
| PGN_1818 | -7.314134563 | -7.381292026 | conserved hypothetical protein |  |
| PGN_1821 | -7.139735514 | -7.943856877 | conserved hypothetical protein |  |
| PGN_1825 | ND | -9.635734442 | hypothetical protein |  |
| PGN_1878 | -6.602706287 | -8.969432572 | conserved hypothetical protein |  |
| PGN_1893 | -7.333817272 | -4.739037979 | hypothetical protein |  |
| PGN_1907 | -7.312429206 | -3.965691949 | conserved hypothetical protein |  |
| PGN_1925 | -6.273329387 | -5.340491058 | conserved hypothetical protein |  |
| PGN_1927 | -5.749239263 | -10.27583933 | conserved hypothetical protein |  |
| PGN_1928 | -6.777524922 | -6.522746289 | conserved hypothetical protein |  |
| PGN_1930 | -7.538561443 | -3.873517937 | conserved hypothetical protein |  |
| PGN_1935 | -3.620703698 | -5.165148193 | hypothetical protein |  |
| PGN_1941 | -8.214678535 | -7.281837321 | putative deoxyribose-phosphate aldolase |  |
| PGN_1942 | -8.451532591 | -6.518692492 | hypothetical protein |  |
| PGN_1948 | -7.011450912 | -8.926604369 | deoxyguanosinetriphosphate triphosphohydrolase |  |
| PGN_1953 | -7.20046708 | -7.067326793 | TonB-dependent outer membrane receptor |  |
| PGN_1960 | -7.263719899 | -8.330881034 | CRISPR-associated protein Cas1 |  |
| PGN_1961 | -7.310594606 | -4.57597785 | CRISPR-associated protein Cas4 |  |
| PGN_1962 | -4.748031796 | -6.329769435 | conserved hypothetical protein |  |
| PGN_1963 | -7.056670226 | -8.123836882 | conserved hypothetical protein |  |
| PGN_1964 | -5.81439661 | -4.474371143 | probable CRISPR-associated helicase Cas3 core |  |
| PGN_1965 | -8.752999409 | -4.296604239 | hypothetical protein |  |
| PGN_1966 | -7.357068093 | ND | conserved hypothetical protein |  |
| PGN_1970 | -6.705342091 | -5.959283303 | arginine-specific cysteine proteinase | rgpA |
| PGN_1975 | -6.890507493 | -8.957662175 | putative regulatory protein |  |
| PGN_1976 | -7.120227508 | -5.427639692 | conserved hypothetical protein |  |
| PGN_1987 | -7.483960845 | -8.957749222 | conserved hypothetical protein |  |
| PGN_1988 | -6.065550771 | -9.717676423 | conserved hypothetical protein |  |
| PGN_1992 | -6.121968591 | -5.488675671 | putative helicase |  |
| PGN_1999 | -7.634607788 | -5.80464733 | putative dihydroorotate dehydrogenase |  |
| PGN_2010 | -5.099758072 | -5.041374417 | putative secreted protein |  |
| PGN_2011 | -7.69981534 | -4.066519781 | putative helicase |  |
| PGN_2016 | -7.452257717 | -8.626336918 | hypothetical protein |  |
| PGN_2017 | -7.660801722 | -7.312928322 | conserved hypothetical protein |  |
| PGN_2025 | -7.364800024 | -6.19491441 | conserved hypothetical protein |  |
| PGN_2035 | -7.995275336 | -6.740509366 | putative peptidase |  |
| PGN_2037 | -7.68505711 | -8.216168665 | DNA-binding protein from starved cells | dps |
| PGN_2042 | -4.98550043 | -8.374574391 | DNA mismatch repair protein MutS |  |
| PGN_2057 | -7.277538484 | -8.792159079 | conserved hypothetical protein |  |
| PGN_2064 | -4.757129951 | -5.966292024 | putative peptidase M48 family |  |
| PGN_2065 | -7.254877445 | -6.706185171 | putative Lys- and Rgp- gingipain domain protein |  |
| PGN_2066 | -7.184895174 | -9.732045288 | putative ABC transporter ATP-binding protein |  |
| PGN_2067 | -6.769388274 | -6.173587199 | TPR domain protein |  |
| PGN_2070 | -7.69075929 | -6.757916651 | conserved hypothetical protein |  |
| PGN_2071 | -6.005310153 | -6.657425643 | probable ketopantoate reductase ApbA/PanE |  |
| PGN_2073 | -7.299244658 | -10.91660055 | conserved hypothetical protein |  |
| PGN_2074 | -7.532963658 | -8.283648649 | conserved hypothetical protein |  |
| PGN_2076 | -7.559667704 | -9.289797586 | conserved hypothetical protein |  |
| PGN_2077 | -7.345786662 | -8.767545559 | conserved hypothetical protein |  |
| PGN_2078 | -5.033775373 | -9.113781228 | conserved hypothetical protein |  |
| PGN_2080 | -5.64757355 | -4.714740071 | conserved hypothetical protein |  |
| PGN_2085 | -7.383937524 | -6.866129069 | putative Fe-S oxidoreductases |  |
| PGN_2088 | -6.475717221 | -6.694880193 | conserved hypothetical protein |  |
| PGN_2089 | -8.377535914 | -4.181659161 | conserved hypothetical protein |  |
| PGN_2090 | -7.202652797 | -7.269818633 | conserved hypothetical protein |  |

^1^ Fold change is represented as a ratio between output and input with negative values indicating a reduction in the output compared to the input pools

^2^ ND: no sequencing reads were detected
