# Supplementary material for: Genes Contributing to Porphyromonas gingivalis Fitness in Abscess and Epithelial Cell Colonization Environments
Source: Front Cell Infect Microbiol. 2017 Aug 28;7:378. doi: 10.3389/fcimb.2017.00378 (PMC5581868; doi:10.3389/fcimb.2017.00378)
Supplement: Supplementary Table 5 — Genes important for fitness only in the TIGK model. [file Table5.docx]

**Supplementary Table 5. Genes important for fitness only in the TIGK model**^1^

| **Feature ID** | **Log2 Fold change** | **Gene description** | **Gene name** |
| --- | --- | --- | --- |
| PGN_0008 | -6.297264797 | ATP-dependent Clp protease ATP-binding subunit ClpC | clpC |
| PGN_0011 | -6.273329387 | conserved hypothetical protein |  |
| PGN_0062 | -5.856388177 | putative conserved protein found in conjugate transposon TraK | traK |
| PGN_0076 | -6.692789835 | putative mobilization protein TraG family |  |
| PGN_0078 | -8.284782467 | hypothetical protein |  |
| PGN_0081 | -5.503603156 | putative Na driven multidrug efflux pump |  |
| PGN_0125 | -5.77665674 | D-isomer specific 2-hydroxyacid dehydrogenase family protein |  |
| PGN_0135 | -6.731101762 | putative H+/peptide symporter |  |
| PGN_0174 | -6.915461146 | probable transcriptional regulator AraC family |  |
| PGN_0211 | -4.840211433 | conserved hypothetical protein |  |
| PGN_0285 | 4.070818637 | pyridine nucleotide-disulphide oxidoreductase |  |
| PGN_0288 | -6.882117226 | conserved hypothetical protein | mfa2 |
| PGN_0291 | -7.091403649 | conserved hypothetical protein | mfa5 |
| PGN_0301 | -6.635681848 | conserved hypothetical protein |  |
| PGN_0317 | -7.285559427 | decarboxylating precorrin-6Y C5,15-methyltransferase | cobL |
| PGN_0318 | -6.543372148 | precorrin-3B C17-methyltransferase | cobH/C |
| PGN_0335 | -3.833294924 | conserved hypothetical protein with Zinc carboxypeptidase domain |  |
| PGN_0340 | -4.432291338 | carboxyl-terminal processing protease |  |
| PGN_0380 | -8.137836494 | partial ROK family transcriptional repressor with glucose kinase domain | glcK |
| PGN_0387 | -7.206955732 | putative O-methyltransferase |  |
| PGN_0482 | -4.806427177 | probable immunoreactive 23 kDa antigen |  |
| PGN_0519 | -6.648508585 | probable competence protein |  |
| PGN_0595 | -6.648738655 | putative conserved protein found in conjugate transposon TraM | traM |
| PGN_0690 | -7.508396959 | conserved hypothetical protein |  |
| PGN_0691 | -5.80944017 | conserved hypothetical protein |  |
| PGN_0728 | -4.249900378 | outer membrane protein 40 precursor |  |
| PGN_0748 | -6.043344505 | gingipain-sensitive ligand A | gslA |
| PGN_0779 | -7.609370838 | probable uracil phosphoribosyltransferase |  |
| PGN_0794 | -6.13948996 | conserved hypothetical protein |  |
| PGN_0856 | -5.18336741 | putative A/G-specific adenine glycosylase |  |
| PGN_0890 | -7.475044339 | putative TonB-dependent outer membrane receptor protein |  |
| PGN_0910 | -6.421155961 | conserved hypothetical protein |  |
| PGN_0925 | -5.923030298 | putative mobilization protein |  |
| PGN_0972 | -6.618547016 | TPR domain protein |  |
| PGN_1013 | -3.800641097 | putative Fe-S oxidoreductase |  |
| PGN_1014 | -6.9058202 | elongation factor G |  |
| PGN_1044 | -4.320629085 | alpha-amylase |  |
| PGN_1076 | -5.923339372 | putative DNA methylase |  |
| PGN_1124 | -6.267573251 | Band 7 protein |  |
| PGN_1261 | -5.716826319 | probable cobalamin adenosyltransferase |  |
| PGN_1328 | -7.267573251 | hypothetical protein |  |
| PGN_1524 | -7.017376341 | conserved hypothetical protein | ptk1 |
| PGN_1525 | -7.091498858 | probable capsular polysaccharide biosynthesis protein | wzb |
| PGN_1539 | -6.965564978 | putative ABC transport system exported protein |  |
| PGN_1629 | -5.339386841 | conserved hypothetical protein with integral membrane domain DUF6 |  |
| PGN_1640 | -6.116156789 | serine/threonine transporter |  |
| PGN_1722 | -5.110655562 | putative uridine kinase |  |
| PGN_1751 | -3.558145159 | DNA primase |  |
| PGN_1752 | -5.229664823 | putative ferredoxin 4Fe-4S |  |
| PGN_1754 | -5.559185866 | hypothetical protein |  |
| PGN_1768 | -6.567317271 | putative DNA-binding response regulator/sensor histidine kinase | gppX |
| PGN_1791 | -8.226600044 | putative flavodoxin |  |
| PGN_1796 | -7.170916513 | conserved hypothetical protein |  |
| PGN_1888 | -6.543372148 | 4-hydroxybutyrate CoA-transferase |  |
| PGN_1903 | -6.476705707 | putative adenine-specific DNA methyltransferase |  |
| PGN_1905 | -6.065636932 | conserved hypothetical protein |  |
| PGN_1932 | -5.410307181 | conserved hypothetical protein |  |
| PGN_2012 | -6.592038371 | outer membrane efflux protein |  |
| PGN_2032 | -6.20867332 | putative alpha-galactosidase |  |

^1^ Fold change is represented as a ratio between output and input with negative values indicating a reduction in the output compared to the input pools
