# Supplementary material for: Genes Contributing to Porphyromonas gingivalis Fitness in Abscess and Epithelial Cell Colonization Environments
Source: Front Cell Infect Microbiol. 2017 Aug 28;7:378. doi: 10.3389/fcimb.2017.00378 (PMC5581868; doi:10.3389/fcimb.2017.00378)
Supplement: Supplementary Table 6 — Genes important for fitness only in the abscess model. [file Table6.docx]

**Supplementary Table 6. Genes important for fitness only in the abscess model**^1^

| **Feature ID** | **Log2 Fold change** | **Gene description** | **Gene name** |
| --- | --- | --- | --- |
| PGN_0026 | -4.052763657 | putative cytidine deaminase |  |
| PGN_0154 | -8.791205224 | conserved hypothetical protein |  |
| PGN_0319 | -6.666628788 | probable RNA polymerase sigma-70 factor ECF subfamily |  |
| PGN_0406 | -6.714025662 | conserved hypothetical protein with glycosyl hydrolase family 92 domain |  |
| PGN_0660 | ND^2^ | putative alkyl hydroperoxide reductase C subunit |  |
| PGN_0710 | -6.536146175 | indolepyruvate ferredoxin oxidoreductase alpha subunit |  |
| PGN_1434 | -7.51195027 | aminoacyl-histidine dipeptidase |  |
| PGN_1435 | -9.59295031 | hypothetical protein |  |
| PGN_1502 | -7.229587923 | conserved hypothetical protein |  |
| PGN_1612 | -9.263766838 | probable beta-phosphoglucomutase |  |
| PGN_1695 | -4.630638446 | putative fructose-bisphosphate aldolase class I |  |
| PGN_1713 | -4.847596102 | conserved hypothetical protein |  |
| PGN_1721 | -4.601755934 | 8-amino-7-oxononanoate synthase | bioF |
| PGN_1929 | -6.719443584 | conserved hypothetical protein |  |

^1^ Fold change is represented as a ratio between output and input with negative values indicating a reduction in the output compared to the input pools

^2^ ND: no sequencing reads were detected
